# PGC-1**α** pathway dysregulation disrupts myofiber specification in a mouse model of SBMA

**DOI:** 10.1172/jci.insight.203215

**Published:** 2026-05-07

**Authors:** Curtis J. Kuo, Laura B. Chopp, Zhigang Yu, Luhan Ni, Hien T. Zhao, Janghoo Lim, Andrew P. Lieberman

**Affiliations:** 1 Department of Pathology,; 2 Cellular and Molecular Biology Graduate Program, Medical Scientist Training Program,; 3 University of Michigan Medical School, Ann Arbor, Michigan, USA.; 4 Department of Genetics, Yale School of Medicine, New Haven, Connecticut, USA.; 5 Ionis Pharmaceutics, Inc., Carlsbad, California, USA.; 6 Department of Neuroscience, Yale School of Medicine, New Haven, Connecticut, USA.

**Keywords:** Muscle biology, Neuroscience, Genetic diseases, Neurodegeneration, Neuromuscular disease

## Abstract

Skeletal muscle pathology is a critical but poorly understood contributor to neuromuscular degeneration in spinal and bulbar muscular atrophy (SBMA), a CAG/polyglutamine (polyQ) expansion disorder caused by mutation in the androgen receptor (AR). Using a gene-targeted SBMA mouse model, we applied single-nucleus RNA sequencing to identify a disease-specific population of skeletal muscle myonuclei that replaced normal myonuclear subtypes. This transition was associated with dysregulation of the pathway governed by PGC-1α, a central regulator of myofiber specification and metabolic identity. PGC-1α dysfunction in SBMA muscle was age, hormone, and polyQ length dependent and was partially rescued by subcutaneous delivery of *AR*-targeted antisense oligonucleotides. Integrated ChIP-seq and RNA-seq analyses revealed that aberrant PGC-1α activity promoted the expression of a distinct set of myofiber specification genes while downregulating those that define healthy Type IIb and Type IIx myonuclei. We propose a model in which this dysfunction arose downstream of polyQ-mediated sequestration of PGC-1α cofactors MEF2, CREB, and CBP, leading to transcriptional reprogramming and cellular dysfunction. These findings implicated PGC-1α dysregulation as a key event linking AR polyQ expansion to skeletal muscle degeneration and suggested a shared mechanism for polyQ-mediated muscle pathology across related neurodegenerative diseases.

## Abstract 2

We demonstrate in SBMA mice a disease-specific population of skeletal muscle myonuclei that replace healthy myonuclear subtypes and reflect dysregulation of the PGC-1 alpha pathway.

## Introduction

Transcriptional dysregulation is a defining feature of the CAG/polyglutamine (polyQ) disorders, a group of age-dependent degenerative diseases in which an expanded Q tract confers proteotoxicity (1). In spinal and bulbar muscular atrophy (SBMA), mutation of the androgen receptor (AR) causes hormone-dependent neuromuscular degeneration in men that is characterized by skeletal muscle weakness and atrophy (2–8). Both nuclear translocation and protein misfolding are critical components of disease pathogenesis, highlighting the importance of impaired nuclear functions (9, 10).

Although early studies of SBMA emphasized lower motor neuron loss, subsequent work has revealed that the disease affects the entire neuromuscular unit. Skeletal muscle pathology has emerged as a key early feature of neuromuscular degeneration in SBMA. In patients, serum creatine kinase, a biomarker of myopathy, is substantially elevated, whereas neuropathy biomarkers such as neurofilament light and heavy chains remain unchanged (6, 11–14). Mouse models that recapitulate the human disease further support a primary role for skeletal muscle in pathogenesis. Gene-targeted AR113Q mice, which carry a humanized *AR* exon 1 containing 113 CAG repeats, exhibit myopathy months before spinal cord involvement (15). Moreover, subcutaneous delivery of *AR*-targeted antisense oligonucleotides (ASOs), which reduce polyQ AR expression in peripheral tissues, substantially mitigates pathology in both muscle and spinal cord (16–18). Complementary findings from conditional BAC fxAR121-transgenic mice demonstrate that deletion of polyQ AR only in skeletal muscle attenuates disease manifestations (19). Collectively, these studies establish skeletal muscle as a critical driver of SBMA pathogenesis and reveal extensive crosstalk between peripheral tissues and the central nervous system.

Physiological analyses of SBMA models have defined multiple features of muscle dysfunction, including reduced contractile force in vivo and ex vivo (20, 21), a metabolic shift toward oxidative phenotypes with selective atrophy of glycolytic fibers (17, 22–24), mitochondrial abnormalities (17, 22, 25), altered NAD^+^ metabolism (26, 27), neuromuscular junction (NMJ) hyperexcitability (20, 21, 28–31), and age-dependent NMJ denervation (18, 32). Despite this detailed characterization of SBMA phenotypes, the molecular mechanisms underlying polyQ AR–mediated muscle dysfunction remain poorly understood.

To define the transcriptional landscape driving SBMA muscle pathology, we performed single-nucleus RNA sequencing (snRNA-seq) on tibialis anterior (TA) muscle from symptomatic AR113Q mice. This analysis revealed a previously unrecognized, disease-specific population of myonuclei accompanied by the loss of canonical myonuclear clusters characteristic of healthy muscle. This change was associated with marked dysregulation in the expression of PGC-1α target genes, including those that mediate healthy fiber type specification. We propose that SBMA muscle pathology is a consequence of PGC-1α pathway dysregulation downstream of the functional sequestration of its canonical binding partners that regulate transcription, and we suggest that this mechanism is shared by other polyQ diseases involving skeletal muscle.

## Results

### snRNA-seq reveals a disease-specific population of myonuclei in aged AR113Q mice.

**Figure 1:**
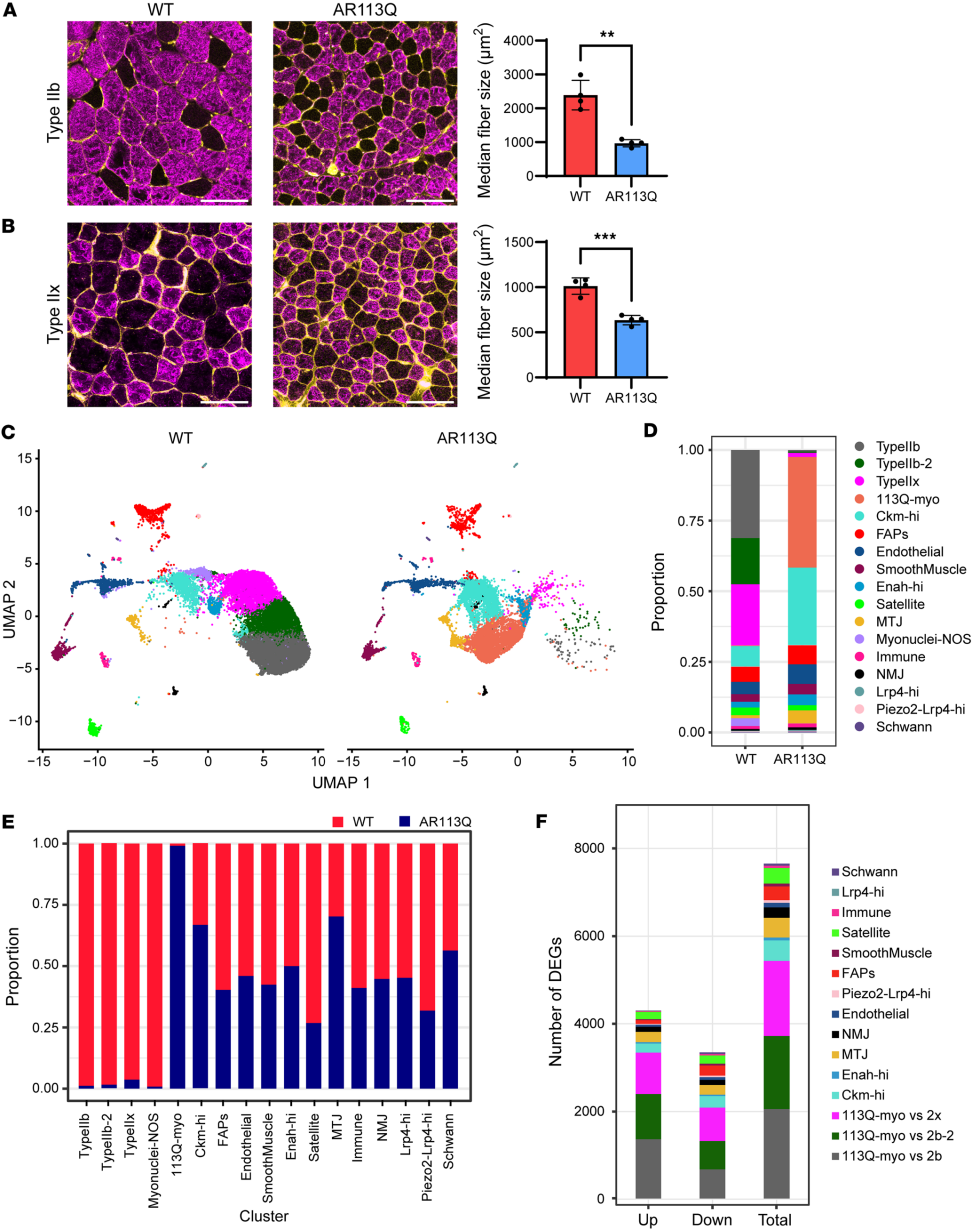
Single-nucleus RNA sequencing reveals a novel, disease-specific population of myonuclei in aged AR113Q mice. (**A** and **B**) Representative immunofluorescence images showing staining of Type IIb (**A**) or Type IIx (**B**) fibers (magenta) and muscle fiber edges (yellow) in tibialis anterior (TA) from WT (left) and AR113Q (right) male mice at 52 weeks. Right: Quantification of the median cross-sectional area of corresponding muscle fibers (*n* = 4 mice/group, total fibers measured = 500–2000 per mouse). Scale bars: 100 μm. (**C**) Unsupervised clustering of nuclei isolated from TA of 3 male WT and 3 male AR113Q mice at 52 weeks, displayed by UMAP. (**D**) Stacked bar graphs showing relative proportion of the population of each nucleus type across all WT (left) or AR113Q (right) nuclei. (**E**) Stacked bar graphs showing distribution of nuclei, for each cluster type, between WT (red) and AR113Q (blue) genotype. (**F**) Number of upregulated, downregulated, and total differentially expressed genes (DEGs) per cluster comparison between WT and AR113Q. For most of the comparisons, DEGs were counted by comparing expression in corresponding clusters, AR113Q vs. WT. However, due to the large discrepancy in number of nuclei in the Type IIb, Type IIb-2, Type IIx, and 113Q-myo clusters, DEGs were counted by pairwise comparisons as indicated. FAPs, fibroadipogenic progenitors; MTJ, myotendinous junction; NOS, not otherwise specified; NMJ, neuromuscular junction. Data are mean ± SD. **P < 0.01, ***P < 0.001 by 2-tailed unpaired *t* test with Welch’s correction. (**A**) *t* = 6.341; df = 3.374; *P* = 0.0055. (**B**) *t* = 7.199, df = 4.837, *P* = 0.0009.

To characterize skeletal muscle deficits in SBMA mice, we analyzed littermate AR113Q and wild-type (WT) males at 52 weeks. At this age, AR113Q males are weak, as measured by grip strength assay, and show atrophy of the TA (15, 18, 33), a disease-relevant hindlimb muscle that is primarily composed of fast-twitch fibers. Analysis of the TA muscle of AR113Q males demonstrated significant atrophy of both Myh4-positive Type IIb fibers (Figure 1A) and Myh1-positive Type IIx fibers (Figure 1B). To further characterize AR113Q skeletal muscle pathology, we performed snRNA-seq on the TA from 3 WT and 3 AR113Q male mice at 52 weeks. After thorough preprocessing and quality control, we integrated the data across individual samples, yielding a total of 27,216 high-quality nuclei, including 9,500 from AR113Q and 17,716 from WT mice, which were sequenced at a depth of approximately 70,000 reads per nucleus (Supplemental Table 1; supplemental material available online with this article; https://doi.org/10.1172/jci.insight.203215DS1). Unsupervised clustering of the data generated 17 unique clusters. We identified cluster-specific genes, and by comparing those genes to previously established marker genes (34), annotated clusters encompassing the major skeletal muscle cell types. We identified 9 distinct clusters of myonuclei (Type IIb, Type IIb-2, Type IIx, Myonuclei-NOS, Ckm-hi, Enah-hi, NMJ associated, myotendinous junction [MTJ] associated, and 113Q-myo). In addition, the dataset included fibroadipose, endothelial, smooth muscle, satellite, immune, Schwann cells, as well as 2 small clusters designated Lrp4-hi and Piezo2-Lrp4-hi (Figure 1, C and D, Supplemental Figure 1, and Supplemental Table 2). Strikingly, the UMAP plot of all nuclei indicated a dramatic change in the myonuclei population of AR113Q muscle. The myonuclei clusters Types IIb, IIb-2, and IIx, which were prominently identified in the WT samples, were largely absent in the AR113Q samples. Instead, an alternative cluster arose, which we termed the 113Q-myo cluster (Figure 1, C and D). Among all nuclei of a given population, the myofiber-associated nuclei (Types IIb, IIb-2, IIx, and 113Q-myo, as well as the small population of Myonuclei_NOS) were dramatically and significantly skewed toward having originated almost exclusively from either WT or AR113Q samples; all other identified clusters had nuclei that were more evenly split between genotypes (Figure 1E, statistics in Supplemental Table 2). To determine the transcriptomic impact of the AR113Q mutation, we identified differentially expressed genes (DEGs) between WT and AR113Q using DESeq2 (35) (Figure 1F). Because of the paucity of Types IIb, IIb-2, and IIx nuclei in AR113Q muscle and the absence of 113Q-myo nuclei in WT muscle, differential expression analyses for these clusters were run using the pairwise comparisons indicated in the legend for Figure 1F. In aggregate, these analyses identified the presence of a disease-specific myonuclei population in AR113Q muscle, with the concomitant loss of the dominant myofiber nuclei clusters that characterize healthy muscle.

### The 113Q-myo cluster is characterized by loss of fiber type specificity.

**Figure 2:**
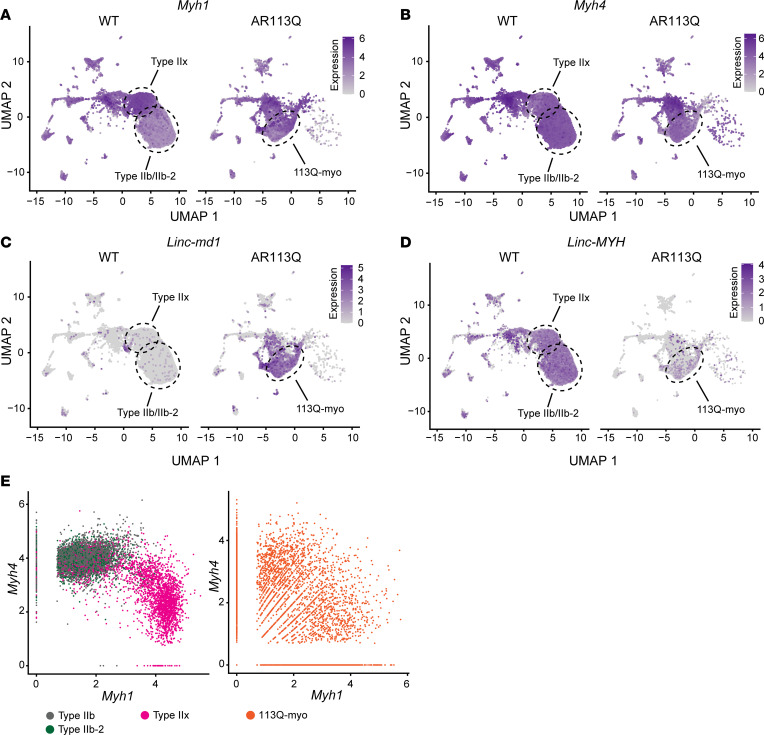
Altered expression of *Myh1* and *Myh4* in AR113Q muscle. (**A**–**D**) Expression of *Myh1* (**A**), *Myh4* (**B**), *Linc-md1* (**C**), and *Linc-MYH* (**D**) over all nuclei in snRNA-seq, separated by WT (left) and AR113Q (right). (**E**) Scatter plots showing relative expression of *Myh4* and *Myh1* for each nucleus among the Type IIb, Type IIb-2, and Type IIx clusters in WT (left) and the 113Q-myo cluster in AR113Q (right).

Having identified a population of nuclei specific to AR113Q muscle, we sought to characterize the gene signature that defined this cluster. Canonically, myonuclei are defined, in part, by the myosin heavy chain gene they predominantly express: *Myh1* for Type IIx and *Myh4* for Type IIb (36). Indeed, the Type IIx cluster in WT muscle was elevated in *Myh1* expression compared with all nuclei (Figure 2A). While *Myh4* was not among the genes that were statistically elevated in the Type IIb and IIb-2 clusters relative to all other nuclei, we posit that its absence among cluster-defining genes for those populations was a consequence of globally high *Myh4* expression throughout our dataset (Figure 2B). Instead of *Myh4*, it was the presence of other top genes identified in these clusters, which matched published annotations, that was used to identify these 2 populations (Figure 1C and Supplemental Table 3). Of the top cluster-defining genes in the 113Q-myo population, the expression of *Linc-md1* (a long noncoding RNA also known as *Gm28653*) was nearly exclusive to nuclei found in AR113Q muscle (Figure 2C). On the other hand, *Linc-MYH* (also known as *2310065F04Rik*), a long noncoding RNA found on the same locus as *Myh1*, *Myh2*, and *Myh4* that helps regulate their expression (37), was mainly expressed in nuclei found in WT muscle (Figure 2D). To more specifically assess the canonical delineation of *Myh4* and *Myh1* as defining genes for Types IIb and IIx myonuclei, we plotted the expression of these 2 genes in all myonuclei (Figure 2E). While WT myonuclei showed a clear distinction in *Myh4* and *Myh1* expression by cluster (Type IIb/IIb-2 vs. Type IIx), this specificity was largely absent in 113Q-myo nuclei, suggesting loss of myofiber specificity in AR113Q muscle.

**Figure 3:**
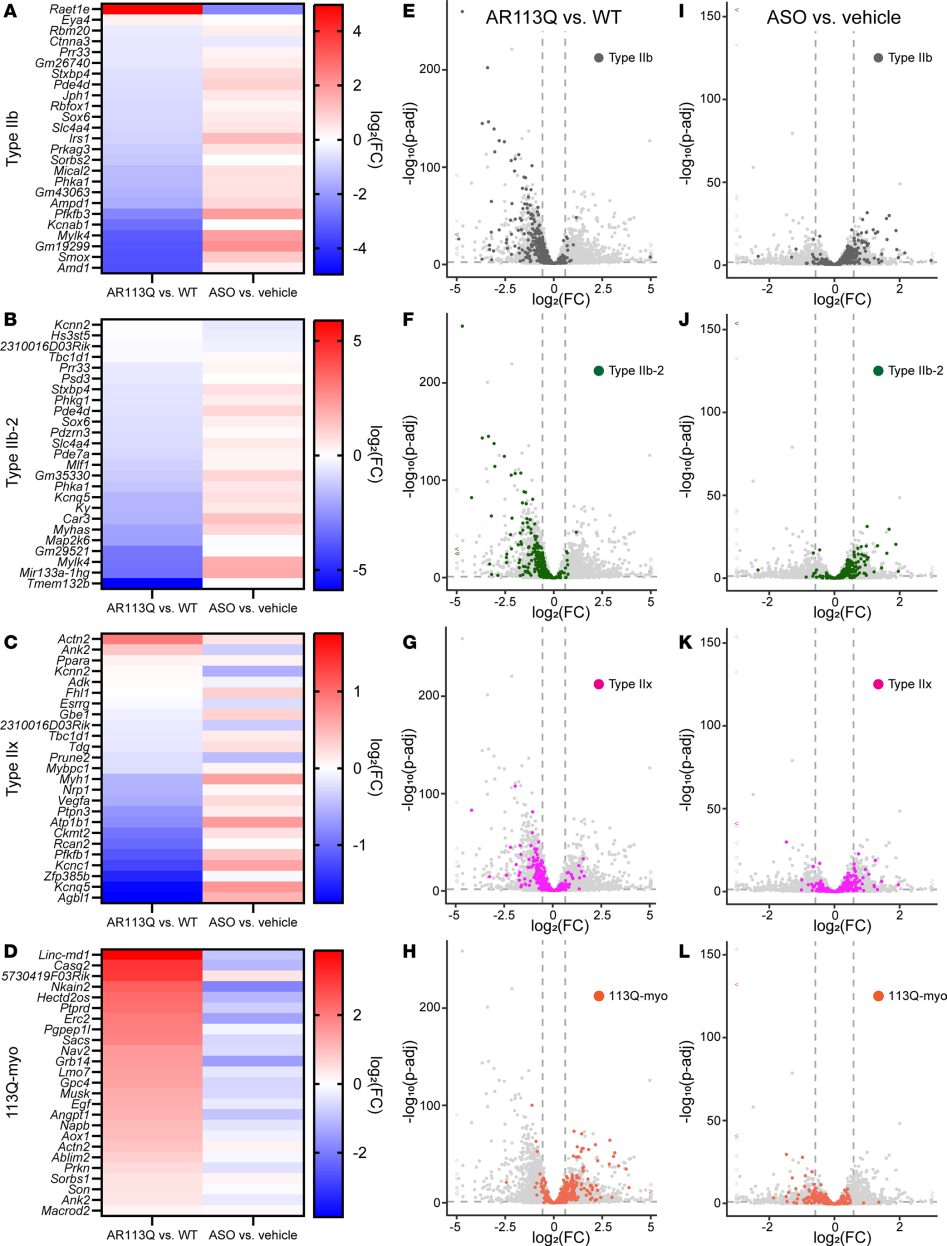
Rescue of myonuclei cluster–defining genes by *AR*-targeted ASO. AR113Q or WT males were administered *AR*-targeted ASO (25 mg/kg body weight) or vehicle subcutaneously, once per week, from 26 to 52 weeks. (**A**–**D**) Heatmaps showing log_2_(fold change) of the top 25 cluster-defining genes for each of the indicated myonuclei groups, as assessed by bulk RNA-seq of TA from independent cohorts of 52-week male mice (*n* = 4/group). Comparisons were between vehicle-treated AR113Q and vehicle-treated WT (left column), and between ASO-treated AR113Q and vehicle-treated AR113Q (right column). Color scales, ranging from red (positive) to blue (negative), were set by mapping the log_2_(FC) with the largest absolute value for each set of 25 genes to the appropriate extreme (pure blue or pure red) and setting the inverse of that value to the other color, such that log_2_(FC) = 0 is exactly in the middle as pure white. (**E**–**L**) Overlay of all cluster-defining genes for the indicated cluster, color coded as in Figure 1C, among the volcano plot of all genes. Dotted lines indicate thresholds of *P*adj < 0.05, |log_2_(FC)| > 0.5849. (**E**–**H**) Vehicle-treated AR113Q vs. vehicle-treated WT. (**I**–**L**) ASO-treated AR113Q vs. vehicle-treated AR113Q.

To further explore the possibility that AR113Q muscle was characterized by a loss of myofiber specificity, we performed bulk RNA-seq on the TA from independent cohorts of AR113Q and WT male mice at 52 weeks. This analysis also included AR113Q males treated from 26 to 52 weeks with subcutaneously administered *AR*-targeted ASOs. As recently reported, this intervention ameliorates several age-dependent degenerative phenotypes of the neuromuscular unit displayed by AR113Q mice that characterize disease progression, including muscle fiber atrophy, NMJ denervation, lower motor neuron soma atrophy, transcriptional changes in spinal cord, and early death (18). Bulk RNA-seq showed that approximately 17% of genes were differentially expressed between AR113Q and WT, and approximately 6% of genes were differentially expressed between ASO-treated and vehicle-treated AR113Q mice (Supplemental Figure 2). We examined changes in the expression of cluster-defining genes, identified by snRNA-seq, for the 3 healthy myonuclei populations (Types IIb, IIb-2, and IIx) and the 113Q-myo cluster. Many of the top 25 cluster-defining genes in the healthy myonuclei populations were downregulated in the comparison of AR113Q versus WT, and these changes were partially rescued (i.e., upregulated) by ASO treatment of AR113Q mice compared with vehicle-treated AR113Q mice (Figure 3, A–C). Correspondingly, most of the top 25 cluster-defining genes in the 113Q-myo population were upregulated in the AR113Q versus WT comparison and partially reversed (downregulated) by ASO treatment (Figure 3D). This pattern held when examining the expression of all cluster-defining genes, not just the top 25 (Figure 3, E–H for AR113Q vs. WT, Figure 3, I–L for ASO vs. vehicle, and Supplemental Table 4). Bulk RNA-seq of TA isolated from WT and AR113Q males at 5 and 26 weeks confirmed that the loss of healthy myonuclei cluster-defining genes and the appearance of 113Q-myo cluster-defining genes was age dependent (Supplemental Figure 3), demonstrating the loss of fiber type specificity with disease progression.

### The 113Q-myo population does not resemble a previously reported population of acutely denervated myonuclei.

Next, we compared the 113Q-myo population to the myonuclei that arise after acute surgical denervation, as reported recently (38). Several genes characterizing the population of myonuclei arising after acute denervation overlapped with genes defining the 113Q-myo cluster. We confirmed statistically significant upregulation of 6 of 8 tested genes in AR113Q muscle by quantitative PCR (qPCR) (Supplemental Figure 4), indicating partial concurrence between the responses to chronic SBMA versus acute denervation. However, unlike in the acute injury response, the 113Q-myo population showed no upregulation of 15-prostaglandin dehydrogenase (15-PGDH), nor did inhibition of 15-PGDH alter the expression of denervation-associated genes (Supplemental Figures 5 and 6). Similarly, the expression of *Maf*, a transcription factor downregulated in acutely denervated fast-twitch fibers (39), was not altered in 113Q-myo. We conclude that the transcriptional profile of 113Q-myo nuclei shares limited features with acute injury, and that SBMA muscle pathology arises through a distinct pathway.

### The PGC-1α pathway is disrupted in AR113Q muscle.

**Figure 4:**
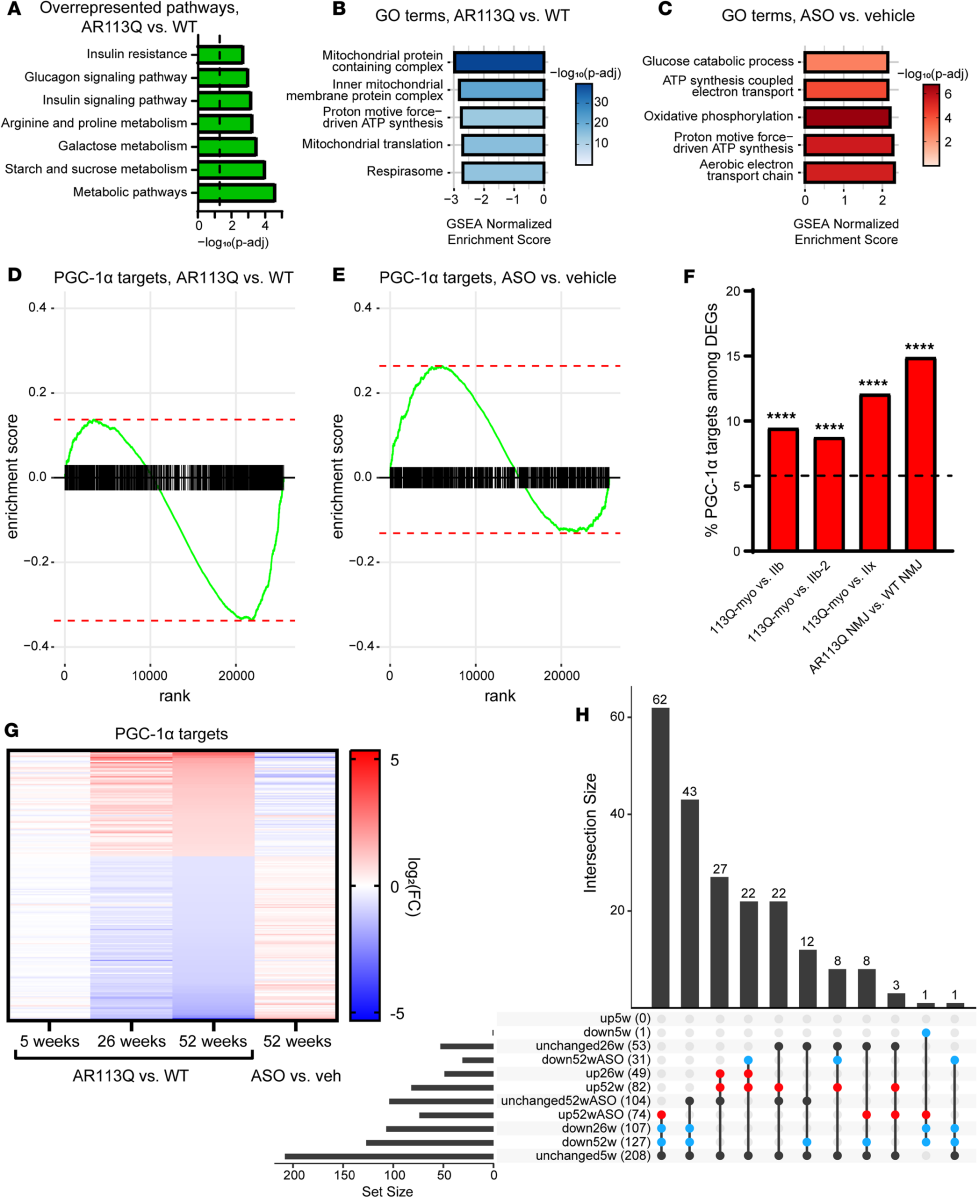
The PGC-1α pathway is disrupted in AR113Q muscle. (**A**)The top overrepresented pathways (by statistical significance) among the DEGs in 52-week AR113Q vs. WT bulk RNA-seq, as identified by iPathway analysis (Advaita Bio, Inc.). (**B** and **C**) Normalized enrichment score and –log_10_(*P*adj) values by GSEA, displayed for the top 5 GO terms downregulated in AR113Q vs. WT (**B**) and upregulated in ASO-treated AR113Q vs. vehicle-treated AR113Q (**C**). (**D** and **E**) GSEA plots showing negative enrichment in AR113Q vs. WT (**D**) and positive enrichment in ASO-treated AR113Q vs. vehicle-treated AR113Q (**E**) of target genes in the PGC-1α pathway (PPARGC1A_TARGET_GENES in MSigDB). (**F**) Percentage of PGC-1α targets among total DEGs in various pairwise comparisons of nuclei established by snRNA-seq (113Q-myo vs. each of the 3 WT myonuclei comparisons, and NMJ nuclei in AR113Q vs. WT). Dotted line indicates the percentage of PGC-1α target genes found in the full snRNA-seq dataset. ****P < 0.0001 for statistically significant overrepresentation of the number of PGC-1α targets found in each of the comparisons, compared with representation among all genes measured by differential abundance analysis, by 2-sided Fisher’s exact test. Raw numbers for contingency analysis are provided in the Supporting Data Values file. (**G**) Heatmap of log_2_(FC) values of the 209 PGC-1α target genes that meet both statistical (*P*adj < 0.05) and biological magnitude (|log_2_[FC]| > 0.5849) thresholds in the 52-week AR113Q vs. WT bulk RNA-seq, and that are present in both 26-week and 5-week AR113Q vs. WT datasets. Comparisons shown: AR113Q vs. WT at 5, 26, and 52 weeks; AR113Q + ASO vs. AR113Q + vehicle at 52 weeks. RNA from *n* = 3 TA in 5-week WT samples and *n* = 4 TA in all other samples. (**H**) UpSet plot visualization of gene groups as shown in **G**. Genes were assigned as “unchanged” when their *P*adj values were ≥ 0.05. Of the remainder, “up” and “down” were assigned by using positive or negative log_2_(FC).

To identify mechanisms underlying SBMA muscle pathology, we performed iPathway analysis (Advaita Bio, Inc.) of the 52-week bulk RNA-seq datasets. This revealed that the list of DEGs in AR113Q versus WT had a significant overrepresentation of genes in pathways related to metabolism (Figure 4A). Gene set enrichment analysis (GSEA) further indicated that the top Gene Ontology (GO) term pathways that were negatively enriched in AR113Q versus WT and positively enriched in ASO-treated versus vehicle-treated AR113Q were related to mitochondria and metabolic function (Figure 4, B and C). GSEA of pathways found in the Gene Transcription Regulation Database (40) revealed that the top negatively enriched transcription factor pathway in AR113Q, compared with WT skeletal muscle, was that of target genes of *Ppargc1a* (*P*adj = 3.04 × 10^–7^, normalized enrichment score = –1.567) (Supplemental Table 5). PGC-1α, encoded by *Ppargc1a*, is a transcriptional coactivator that acts as a central metabolic regulator (41). In addition to its well-studied function of inducing mitochondrial biogenesis in response to tissue metabolic demand, PGC-1α also impacts myofiber type determination in skeletal muscle (42). GSEA revealed that genes in the PGC-1α pathway were negatively enriched in AR113Q versus WT DEGs and rescued in ASO versus vehicle DEGs (Figure 4, D and E). By snRNA-seq, PGC-1α target genes were also overrepresented among all DEGs in pairwise comparisons of the 113Q-myo versus Types IIb, IIb-2, and IIx clusters, as well as the NMJ-associated nuclei in the comparison of AR113Q versus WT samples (Figure 4F).

To examine the age dependence of this phenotype, we analyzed bulk RNA-seq of the TA at 2 earlier ages: 26 weeks, an age with substantial muscle atrophy but minimal NMJ or spinal cord pathology, and 5 weeks, an age prior to symptom onset (15, 18, 33). Principal component analysis (PCA) and volcano plots at 26 weeks and 5 weeks reflected the presence and absence of large-scale gene expression differences between AR113Q and WT mice, respectively (Supplemental Figure 7 and Supplemental Table 6). Among all DEGs at 52 weeks, 209 were PGC-1α target genes that were also measured at both earlier ages. These PGC-1α targets showed age-dependent dysregulation that was exacerbated with disease progression and rescued by treatment with *AR*-targeted ASO (Figure 4, G and H), confirming this as a degenerative rather than developmental phenotype.

**Figure 5:**
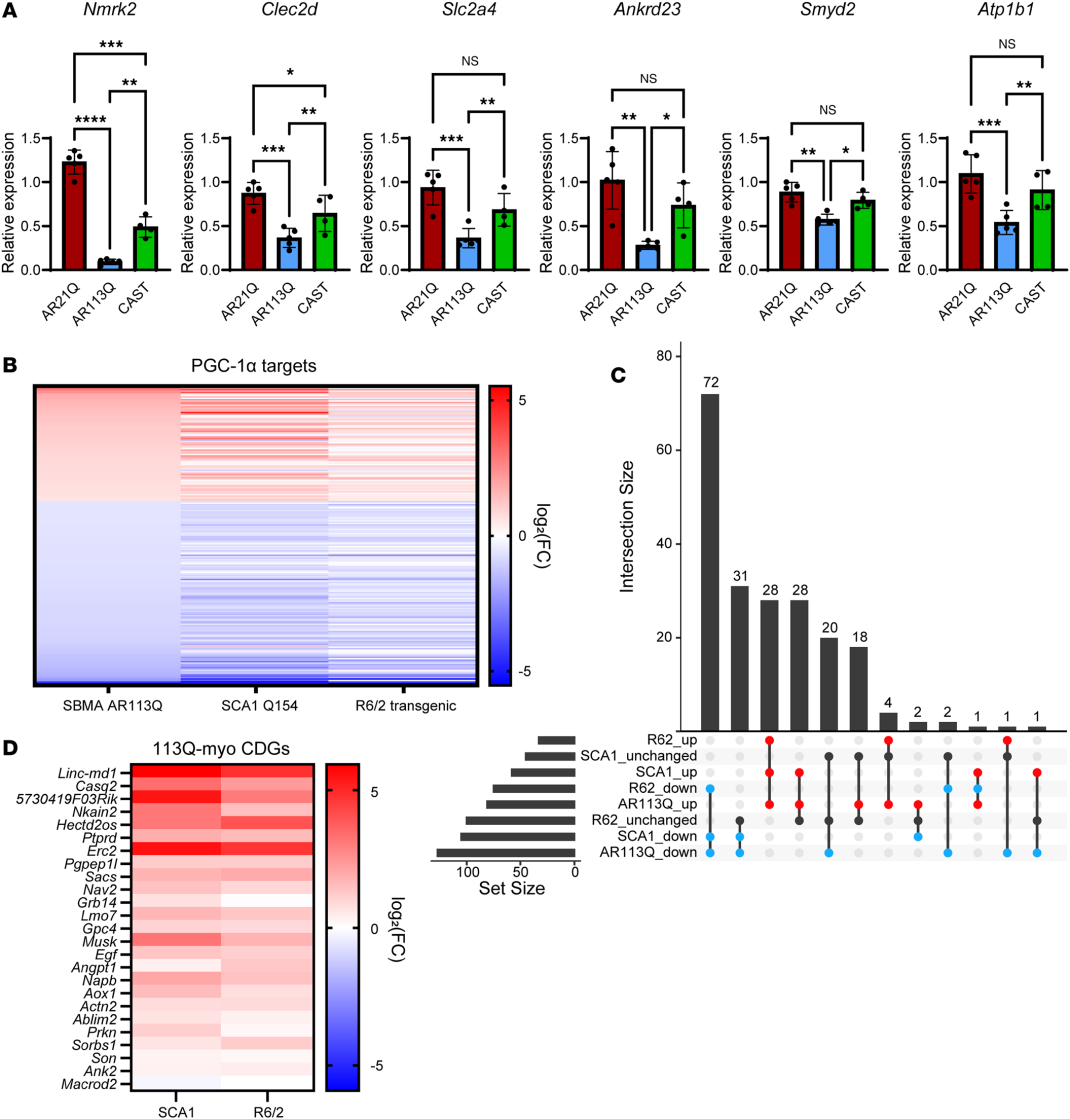
PGC-1α pathway dysfunction is hormone and glutamine tract length dependent in AR113Q mice and is conserved in 2 other polyQ mouse models. (**A**) Relative gene expression by qPCR of 6 PGC-1α target genes in the TA of male mice at 52 weeks from independent cohorts of AR21Q, AR113Q, and AR113Q castrated (“CAST”) mice (*n* = 5/group for AR21Q and AR113Q, *n* = 4 for the castrated group). (**B**) Heatmap of log_2_(FC) values of the PGC-1α target genes studied in Figure 4G. RNA from TA of AR113Q and WT males at 52 weeks (*n* = 4/genotype), TA of SCA1 Q154 and WT males at 24 weeks (*n* = 3/genotype), and quadriceps of R6/2 transgenic and WT males at 12 weeks (*n* = 4/genotype). The SBMA AR113Q column is repeated from Figure 4G for ease of comparison. (**C**) UpSet plot visualization of overlap between PGC-1α target genes with differential expression in AR113Q, SCA1 Q154, and R6/2 transgenic skeletal muscle from **B**. Genes were assigned as “unchanged” when their *P*adj values were ≥ 0.05 or when |log_2_(FC)| < 0.5849, “up” when *P*adj < 0.05 and |log_2_(FC)| > 0.5849, and “down” when *P*adj < 0.05 and |log_2_(FC)| < 0.5849 in the comparisons of polyQ disease to their corresponding age-matched WT controls. (**D**) Heatmap of |log_2_(FC)| values of the top 25 cluster defining genes for the 113Q-myo population as seen in Figure 3D, among disease vs. WT bulk RNA-seq analyses as in **B**. While indications for statistical significance are not shown here, they are included in the accompanying Supporting Data Values file; 21 out of 25 genes met thresholds for differential expression in both SCA1 Q154 and R6/2 transgenic disease vs. WT. Order of genes is as shown in Figure 3D. Data are mean ± SD. NS, not significant. *P < 0.05; **P < 0.01; ***P < 0.001; ****P < 0.0001 by 2-way ANOVA with Tukey’s multiple-comparison test (**A**). All results of statistical analyses are in left-to-right order (*Nmrk2*, *Clec2d*, *Slc2a4*, *Ankrd23*, *Smyd2*, *Atp1b1*). (**A**) Main ANOVA results: *F* = 103.9, 38.19, 30.38, 17.98, 14.93, 32.70; df = 2; *P* = <0.0001, 0.0002, 0.0004, 0.0017, 0.0030, 0.0003. For *P*adj values by multiple comparisons testing, see the Supporting Data Values file.

We further characterized PGC-1α pathway dysregulation using AR21Q mice. Like AR113Q, AR21Q mice were generated by replacing much of mouse *Ar* exon 1 with the first exon of human *AR,* encompassing the CAG repeat (43). However, these mice express a polyQ tract that is of non-pathogenic length, and they are phenotypically identical to WT mice. qPCR showed that the relative expression of 6 PGC-1α target genes was downregulated in AR113Q versus AR21Q TA at 52 weeks (Figure 5A). Moreover, surgical castration prior to sexual maturity partially or fully rescued the expression of PGC-1α target genes in AR113Q males (Figure 5A). These results indicated a dependence of PGC-1α pathway dysregulation on both length of the polyQ tract and presence of endogenous androgens.

We expanded this analysis to 2 other mouse models of polyQ repeat diseases with skeletal muscle involvement: the Q154-knockin mouse model of spinocerebellar ataxia type 1 (SCA1), with a CAG repeat expansion in the *Atxn1* gene (44, 45), and the R6/2 transgenic mouse model of Huntington disease (HD), with a CAG repeat expansion in a fragment of the *HTT* gene (46, 47). Using skeletal muscle from male mice of both strains, obtained at time points with similar symptomatic burden as aged AR113Q mice (TA at 24 weeks in SCA1 Q154, quadriceps at 12 weeks in R6/2), we observed overlapping dysregulation of PGC-1α target genes by bulk RNA-seq (Figure 5, B and C, Supplemental Figure 8, and Supplemental Table 7). This suggests that PGC-1α pathway dysregulation was not unique to SBMA, but instead reflects a conserved pathogenic mechanism shared by other polyQ repeat expansion diseases with skeletal muscle involvement. In addition, examining the top 25 cluster defining genes for the 113Q-myo population in these other polyQ models showed that the majority of the top genes (20/25) met the thresholds for significant upregulated expression (*P*adj < 0.05 and log_2_[FC] > 0.5849) in both SCA1 and R6/2 samples (Figure 5D). These data suggest that transcriptional dysregulation of myonuclei in these 2 other polyQ models with skeletal muscle involvement may be similar to that which occurs in AR113Q mice.

### Altered binding of PGC-1α to promoters of myonuclei specification genes.

**Figure 6:**
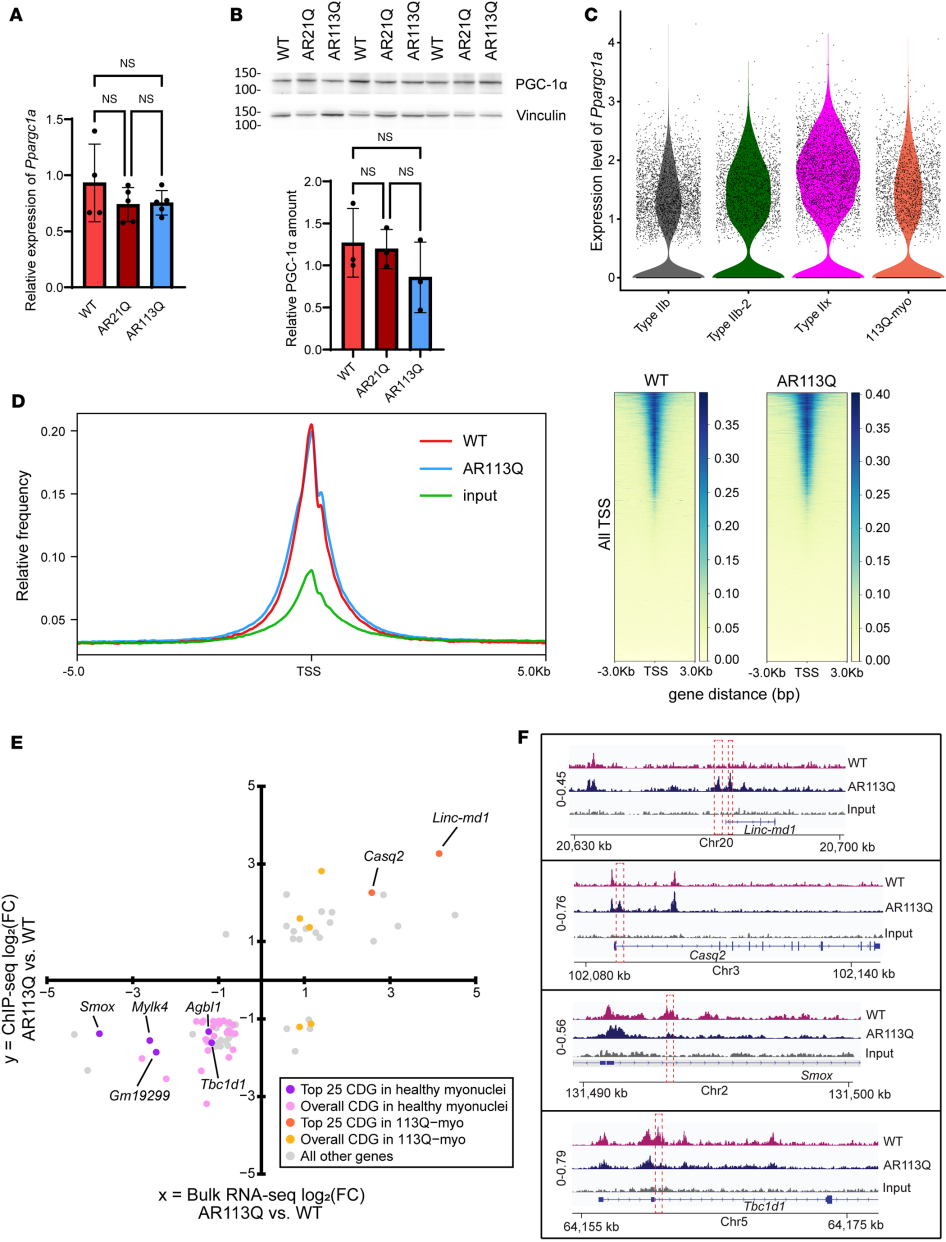
PGC-1α binding to promoters of myonuclei specification genes is altered in AR113Q muscle. (**A**) Relative gene expression by qPCR of *Ppargc1a* mRNA in WT, AR21Q, and AR113Q TA at 26 weeks (*n* = 4 for the WT group, *n* = 5/group for AR21Q and AR113Q). (**B**) Relative amount of PGC-1α protein, normalized to vinculin, in WT, AR21Q, and AR113Q TA at 26 weeks (*n* = 3/group). (**C**) Expression of *Ppargc1a* in Type IIb, Type IIb-2, Type IIx, and 113Q-myo myonuclear clusters defined in 52-week AR113Q and WT males by snRNA-seq. (**D**–**F**) PGC-1α ChIP-seq was performed on nuclei isolated from the TA of WT and AR113Q males at 26 weeks (*n* = 4 WT, 7 AR113Q). (**D**) ChIP-seq results of average occupancy of PGC-1α near known transcription start sites (TSSs) in chromatin extracted from WT and AR113Q TA at 26 weeks (total of 200 mg pooled muscle per genotype). (**E**) Genes that met magnitude and statistical thresholds in both ChIP-seq differential analysis and bulk RNA-seq of independent cohorts of mice, all obtained from AR113Q vs. WT TA at 26 weeks (ChIP-seq, |log_2_[FC]| > 1 and FDR < 0.05, *n* = 4 WT, 7 AR113Q; bulk RNA-seq, |log_2_[FC] > 0.5849 and *P*adj < 0.05, *n* = 4/group). Genes color coded by their presence in the lists of cluster-defining gene (CDG) sets from snRNA-seq data (Figure 1-3): light purple if present in one or more of the Types IIb, IIb-2, and IIx CDG sets, light orange if present in the 113Q-myo CDG set, dark purple/orange if present among the top 25 in any corresponding CDG set. (**F**) Integrative Genomics Viewer snapshots demonstrating differential enrichment of PGC-1α at promoter regions (highlighted by dashed boxes). *Linc-md1* and *Casq2* were 2 of the top 25 CDGs for the 113Q-myo cluster. *Smox* and *Tbc1d1* were 2 of the top 25 CDGs in the IIb, IIb-2, and/or IIx clusters. Data are mean ± SD. NS, not significant by 2-way ANOVA with Tukey’s multiple-comparison test. (**A**) Main ANOVA results: *F* = 1.632; df = 2; *P* = 0.2620; Multiple comparisons: WT vs. AR21Q *P*adj = 0.2831; WT vs. AR113Q *P*adj = 0.3322; AR21Q vs. AR113Q *P*adj = 0.9893. (**B**) Main ANOVA results: *F* = 1.077; df = 2; *P* = 0.4224; Multiple comparisons: WT vs. AR21Q *P*adj = 0.9665; WT vs. AR113Q *P*adj = 0.4322; AR21Q vs. AR113Q *P*adj = 0.5491.

We next sought to identify the mechanism governing PGC-1α pathway dysregulation in SBMA. We found no significant differences in 52-week WT, AR21Q, and AR113Q TA expression of the *Ppargc1a* gene at either the mRNA level (Figure 6A) or the protein level (Figure 6B). In addition, snRNA-seq analysis of myonuclei clusters showed no significant difference in *Ppargc1a* expression between the 113Q-myo cluster and the 3 healthy myonuclei clusters (Figure 6C). *Ppargc1a* expression was also unchanged in SCA1 Q154 and R6/2 skeletal muscle (Supplemental Figure 9).

We considered the possibility that PGC-1α may be functionally sequestered by misfolded polyQ proteins, impairing its normal role in the regulation of gene expression as a transcriptional coactivator. We assessed chromatin binding of PGC-1α by performing chromatin immunoprecipitation sequencing (ChIP-seq) using AR113Q and WT TA at 26 weeks, an age at which substantial muscle pathology is present in this model (18). Interestingly, this showed no global change in chromatin occupancy near transcription start sites (TSSs), suggesting that there was not widespread loss of PGC-1α function in AR113Q muscle (Figure 6D). We then identified genes with differential peak binding within 5 kb of their TSS and compared these to results of bulk RNA-seq at 26 weeks. This yielded 76 genes that met thresholds for magnitude and statistical significance in both analyses (for ChIP-seq, |log_2_[FC]| > 1 and FDR < 0.05; for bulk RNA-seq, |log_2_[FC]| > 0.5849 and *P*adj < 0.05), indicating that, for these genes, differential gene expression correlated with differential PGC-1α targeting for transcriptional coactivation in AR113Q versus WT muscle (Figure 6E). Most genes were consistent in their direction of significant change, with 49 genes downregulated in expression in conjunction with decreased PGC-1α chromatin binding near their TSS, while 20 genes were upregulated with increased chromatin binding. Intriguingly, about half of these genes were found to be myonuclei cluster-defining genes identified in the snRNA-seq dataset (Figures 1 and 3, and Supplemental Table 3). Several genes were also among the top 25 in one or more sets of cluster-defining genes for Types IIb, IIb-2, IIx, or 113Q-myo (Figure 6, E and F). Notably, 29 cluster-defining genes for healthy myonuclei exhibited both decreased expression and PGC-1α chromatin binding in AR113Q muscle, while 5 113Q-myo cluster-defining genes showed increased expression and chromatin binding. We performed GO term analysis on the genes in the decreased chromatin binding–decreased expression quadrant (Figure 6E, lower left), and although the total number of genes was modest, the muscle-related GO terms sarcomere, myofibril, and contractile muscle fiber reached statistical significance (Supplemental Table 8). Taken together, these results suggest that the dramatic shift in myonuclear population in AR113Q muscle, seen by snRNA-seq (Figure 1), was due in part to PGC-1α acting as a transcriptional coactivator for an alternate set of myonuclei specification genes.

**Figure 7:**
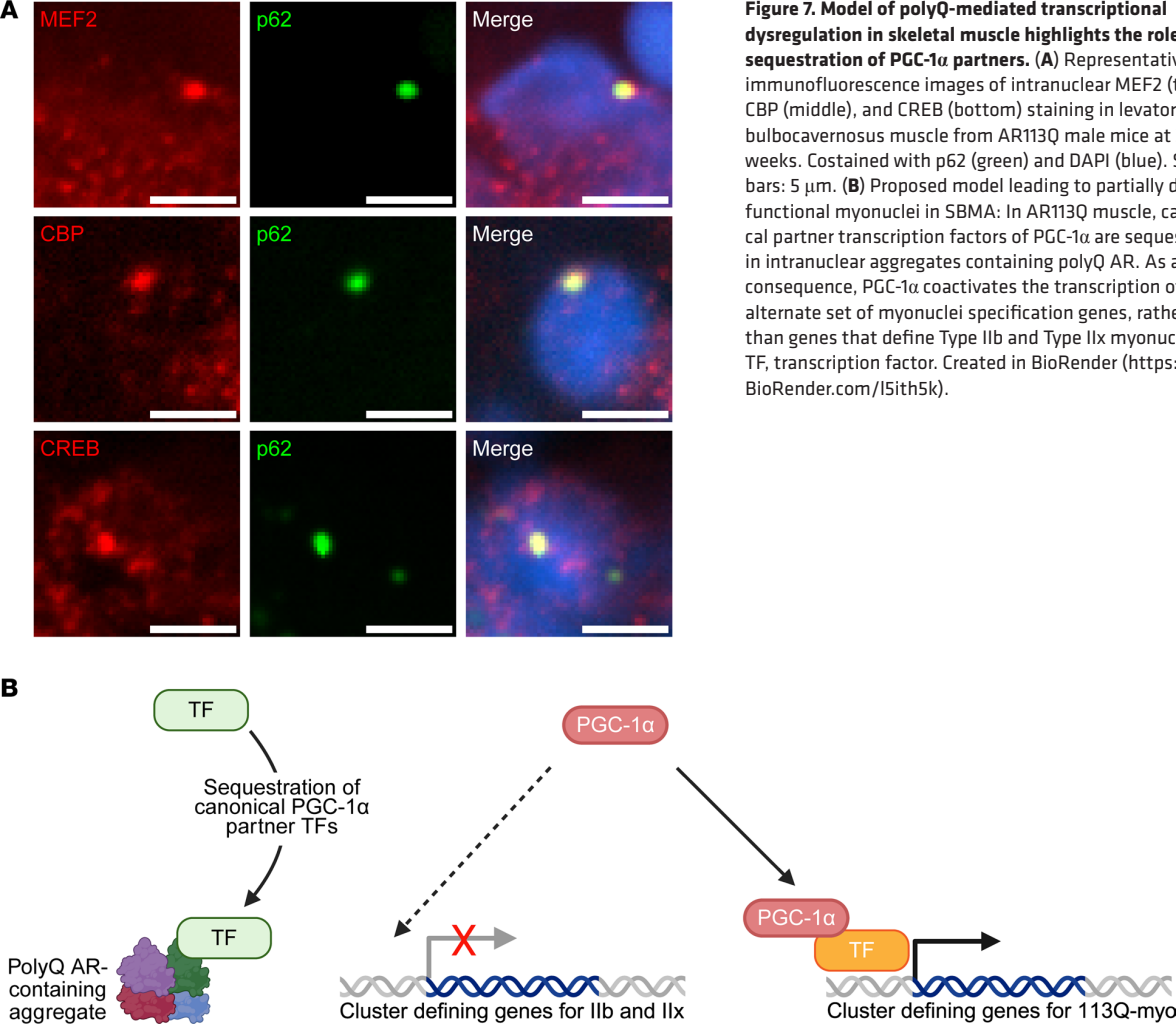
Model of polyQ-mediated transcriptional dysregulation in skeletal muscle highlights the role of sequestration of PGC-1α partners. (**A**) Representative immunofluorescence images of intranuclear MEF2 (top), CBP (middle), and CREB (bottom) staining in levator ani/bulbocavernosus muscle from AR113Q male mice at 26 weeks. Costained with p62 (green) and DAPI (blue). Scale bars: 5 μm. (**B**) Proposed model leading to partially dysfunctional myonuclei in SBMA: In AR113Q muscle, canonical partner transcription factors of PGC-1α are sequestered in intranuclear aggregates containing polyQ AR. As a consequence, PGC-1α coactivates the transcription of an alternate set of myonuclei specification genes, rather than genes that define Type IIb and Type IIx myonuclei. TF, transcription factor. Created in BioRender (https://BioRender.com/l5ith5k).

Several transcriptional regulators that are coactivated by PGC-1α, including myocyte enhancer factor 2 (MEF2), cAMP response element binding (CREB), and CREB-binding protein (CBP), are sequestered by proteins with polyQ tracts, and these transcription factors are functionally impaired in models of SBMA, HD, and SCA1 (48–53). Using the levator ani/bulbocavernosus muscle, which is an androgen-sensitive skeletal muscle in male mice that highly expresses AR, resulting in frequent polyQ AR and p62-positive intranuclear inclusions (48), we confirmed the sequestration of MEF2, CBP, and CREB into intranuclear inclusions in AR113Q muscle (Figure 7A) and the lack of such sequestration in WT muscle (Supplemental Figure 10). In AR113Q muscle, approximately 60% of p62-positive intranuclear inclusions stained positively for each of these transcriptional regulators. In contrast, overexpression of the polyQ AR had no effect on the intranuclear distribution of PGC-1α in a cellular model (Supplemental Figure 11), consistent with its intact chromatin association (Figure 6D) and function as a coactivator for an alternate set of myofiber specification genes in AR113Q muscle (Figure 6E, F). Among these alternatively activated genes was *Linc-md1*, the top cluster-defining gene for 113Q-myo (Figures 1–3) and one of the ChIP-validated targets of PGC-1α with increased chromatin binding and increased gene expression (Figure 6, E and F). In addition to its elevation in the 113Q-myo cluster, expression of *Linc-md1* was increased in AR113Q bulk RNA-seq relative to WT (Supplemental Figure 12A) as well as in SCA1 Q154 and R6/2 skeletal muscle (Supplemental Figure 12, B and C). These observations suggest the following model of polyQ-mediated muscle pathology (Figure 7B): polyQ-containing proteins sequester and functionally impair transcription factors normally coactivated by PGC-1α. In models of SBMA, HD, and SCA1, this results in diminished function of MEF2, CBP, and CREB (48, 50, 52, 53). As a consequence, instead of maintaining transcription of the cluster-defining genes for Types IIb, IIb-2, and IIx myonuclei as in healthy muscle, in AR113Q muscle, PGC-1α coactivates the transcription of an alternate set of myonuclei differentiation genes, resulting in diseased muscle.

## Discussion

Our analysis of SBMA muscle defines a paradigm for polyQ-mediated transcriptional dysregulation. We demonstrate that sequestration of transcriptional regulators by disease-causing proteins, such as the polyQ AR, is accompanied by a “second hit” of coactivator dysfunction that amplifies transcriptional dysregulation. The result is coactivator-triggered expression of an alternate set of genes that underlie cellular dysfunction. We experimentally demonstrate this pathogenic cascade in AR113Q skeletal muscle, in which PGC-1α dysregulation underlies critical changes in gene expression downstream of functional sequestration of transcriptional regulators, including MEF2, CBP, and CREB. The consequence is the appearance of what we believe is a novel, disease-specific population of myonuclei, replacing populations of well-defined healthy myonuclei. Underlying this dramatic shift is dysregulation of the PGC-1α pathway, which ordinarily maintains myofiber specification in skeletal muscle along with its function as a central regulator of metabolism (42). Comparison of individual nuclear populations reveals that PGC-1α targets are disproportionately represented among DEGs in NMJ- and myofiber-associated myonuclei. PGC-1α itself is not differentially expressed in WT versus AR113Q muscle at either the protein or mRNA level, nor does it exhibit a global loss of chromatin binding. Instead, and consistent with our model of pathogenesis, integrated ChIP-seq and bulk RNA-seq analyses show upregulation of an alternate set of myofiber specification genes for the 113Q-myo population, along with downregulation of cluster defining genes for healthy Type IIb and Type IIx myonuclei.

Hallmarks of this pathogenic cascade are also observed in skeletal muscle from R6/2 and SCA1 Q154 mice, indicating that this mechanism may contribute to skeletal muscle pathology in several polyQ disorders. Bulk RNA-seq analysis of skeletal muscle from symptomatic R6/2 and SCA1 Q154 mice shows similar dysregulation of genes in the PGC-1α pathway and increase in 113Q-myo cluster–defining genes. Notably, among the 113Q-myo cluster–defining genes is *Linc-md1*, a long noncoding RNA that governs injury response by activating the transcription factor MEF2 (54). However, as previously described, MEF2 is functionally impaired in SBMA skeletal muscle and in other mouse models of polyQ disease (48). We suggest that the observed myonuclear shift in SBMA skeletal muscle is a consequence of a failed injury response, triggered by altered gene regulation by PGC-1α and the cooccurring functional impairment of MEF2 (Figure 7B).

As the nexus between skeletal muscle and motor neurons, the NMJ was an initial focus of our inquiry. We expected to find an accentuation of pathology at this site of communication between atrophic skeletal muscle and degenerating motor neurons in AR113Q mice; unexpectedly, this was not the case. The NMJ myonuclei were not one of the clusters that had a statistically significant difference in total number of nuclei between WT and AR113Q (Supplemental Table 2). When comparing NMJ-specific gene expression, 235 genes were differentially expressed between WT and AR113Q NMJ myonuclei, a modest sum representing about 1% of all measured genes in snRNA-seq. This is roughly on par with many of the other nuclei comparisons (Figure 1E) and an order of magnitude smaller than the numbers of DEGs between the 113Q-myo nuclei compared with each of the 3 main healthy myonuclei populations (roughly 1600–2000 genes each). Despite the relatively modest number of DEGs in NMJ myonuclei, this group had a statistically significant overrepresentation of PGC-1α targets (Figure 4F), reflecting the occurrence of pathological changes without the dramatic transcriptional remodeling observed in other myonuclei populations.

PGC-1α is expressed in other energetically intensive tissues in addition to skeletal muscle, and it has been implicated in the pathogenesis of HD as a mediator of metabolic deficits in the brain, linking polyQ expansion to mitochondrial dysfunction (55, 56). We examined this pathway in SBMA spinal cord using a recently published dataset of lumbar spinal cord snRNA-seq in AR113Q mice at 52 weeks (18). While PGC-1α targets were expressed in the *ChAT*-positive population of lower motor neurons, they were not significantly overrepresented among DEGs, suggesting that this pathway is not a key component of the transcriptional dysregulation in lower motor neurons. However, other metabolic pathways may be dysregulated in motor neurons due to MEF2C functional impairment, separate from the PGC-1α–mediated skeletal muscle–specific cascade demonstrated here. Recently, MEF2C knockdown in cortical layer V in WT mice was shown to lead to mitochondrial dysfunction, upper motor neuron damage, and resultant behaviors resembling phenotypes seen in mouse models of amyotrophic lateral sclerosis (57).

In conclusion, our findings reveal a muscle-intrinsic mechanism of disease in SBMA in which the polyQ AR sequesters essential transcriptional cofactors, leading to PGC-1α pathway dysregulation, myonuclear reorganization, and an impaired injury response. Because both PGC-1α dysregulation and MEF2 impairment occur in other polyQ models with muscle involvement, we propose that this transcriptional cascade represents a shared mechanism underlying muscle degeneration across polyQ expansion disorders.

## Methods

Further information can be found in Supplemental Methods.

### Sex as a biological variable

As SBMA is an X-linked, sex-limited disorder that affects only males with the mutant allele, only male mice were used in this study.

### Animal studies

AR21Q and AR113Q mice were generated using gene targeting to insert the human *AR* sequence with 21 or 113 CAG repeats, respectively, into mouse *Ar* exon 1, as previously described (15, 33). Animals were backcrossed to C57BL/6J (JAX, strain 000664) for more than 10 generations. All AR113Q male mice used in these studies had a CAG repeat length of at least 97. Mice were kept in climate-controlled cages under a 12-hour light-dark cycle, in accordance with the University of Michigan Unit for Laboratory Animal Medicine. The following genotyping primers were used: 113Q primer pair: forward, 5′-CCAGAATCTGTTCCAGAGCGTG-3′ (MilliporeSigma, 6-FAM labeled); reverse, 5′-TGTTCCCCTGGACTCAGATG-3′ (Invitrogen). 21Q primer triplet: forward, 5′-GAATCTGTTCCAGAGCGTG-3′ (Invitrogen); human reverse, 5′-TGCCCCCTAAGTAATTGTCC-3′ (Invitrogen); mouse reverse, 5′-AGCTGAGTCATCCTGATCTG-3′ (Invitrogen). For ASO experiments, male mice were subcutaneously administered 25 mg/kg *AR*-targeted ASO or sterile PBS (vehicle), once weekly between weeks 26 and 52 of age, as previously described (18). ASOs were made of a 16-mer, 4-8-4 constrained mixed cEt/MOE gapmer with full phosphorothioate backbone modification containing the following sequence: AAGTTGTAGTAGTCGC, which is complementary to human and mouse *AR* transcripts, as detailed previously (18). The ASO was conjugated to palmitic acid via a phosphodiester linkage at the 5′ end. Orchiectomy was performed on AR113Q males at 5–6 weeks, as previously described (15). Euthanasia for the purposes of tissue extraction at experimental end points was performed humanely by isoflurane drop jar, followed by decapitation.

SCA1 Q154 (JAX, strain 005601) mice (44) were maintained on a pure C57BL/6J (JAX, strain 000664) background. The following PCR primers were used for genotyping: forward, 5′-ACCTTCCAGTTCATTGGGTC-3′ (Sigma-Aldrich); reverse, 5′-GCTCTGTGGAGAGCTGGA-3′ (Sigma-Aldrich). Tissue from R6/2 mice (gift from Gillian Bates, University College London, London, United Kingdom) was previously described (48). To confirm sex of mice using harvested tissue, the following PCR primers were used: forward, 5′-CACCTTAAGAACAAGCCAATACA-3′ (Invitrogen); reverse, 5′-GGCTTGTCCTGAAAACATTTGG-3′ (Invitrogen); XX band: 269 bp; XY bands: 269 bp, 353 bp (58).

### Immunofluorescence microscopy

For fiber type staining, TA was harvested from 52-week-old male mice (4 WT, 4 AR113Q), embedded in optical cutting temperature (OCT) compound, and frozen in prechilled isopentane. Frozen sections were obtained at a thickness of 10 μm using a Leica CM1900 Cryostat (Leica Biosystems). A hydrophobic barrier was established around samples using an ImmEdge pen (Vector Labs, H-4000). Samples were rinsed with three 5-minute PBS washes, followed by pretreatment in 0.2% Triton X-100 in PBS (PBS-T) for 5 minutes, and 3 more 5-minute PBS washes. Samples were then blocked for 30 minutes at room temperature with Mouse On Mouse (M.O.M.) Ig Blocking Reagent (Vector Labs, BMK-2202), rinsed with three 5-minute PBS washes, and incubated for 5 minutes in a solution of 1:12.5 M.O.M. Protein Concentrate (Vector Labs, BMK-2202). Primary antibodies diluted in 0.1% PBS-T were then applied overnight at 4°C. After three 5-minute PBS washes, secondary antibodies diluted in 0.1% PBS-T were applied for 1 hour at room temperature and then rinsed off with three 5-minute PBS washes. Wheat germ agglutinin stock solution (1 mg/mL) was diluted to a working concentration of 5 μg/mL in PBS, applied to the samples for 10 minutes at room temperature, and rinsed off with three 5-minute PBS washes. Slides were mounted using VECTASHIELD Antifade Mounting Medium (Vector Labs, H-1000) and imaged using a Nikon A1R confocal microscope. Images were quantified for fiber size and identification of Type IIb and Type IIx fibers using MyoSight (59).

For colocalization studies, levator ani/bulbocavernosus was harvested from 26-week-old male mice (3 WT, 3 AR113Q), embedded, and sectioned, as above. A hydrophobic barrier was established around samples using an ImmEdge pen (Vector Labs, H-4000). Samples were fixed for 4 minutes in ice-cold methanol and permeabilized with three 5-minute washes in 0.1% PBS-T. Samples were blocked for 1 hour at room temperature with 10% normal goat serum in PBS and incubated overnight in primary antibodies diluted in blocking solution at 4°C. After three 5-minute PBS washes, secondary antibodies diluted in blocking solution were applied for 1 hour at room temperature and then rinsed off with three 5-minute washes with 0.1% PBS-T. Slides were mounted with VECTASHIELD Antifade Mounting Medium with DAPI (Vector Labs, H-1200-10) and imaged using a Nikon X1 Yokogawa Spinning Disk confocal microscope.

For all microscopy, image manipulation for figure preparation was limited to generation of maximum projections from *z*-scans, cropping, and linear adjustments of brightness and/or contrast applied equally across entire images in Fiji (60).

### snRNA-seq

TA was harvested from 52-week-old male mice (3 WT, 3 AR113Q) and flash-frozen in liquid nitrogen. Nuclei were extracted with the 10X Chromium Nuclei Isolation kit (10X Genomics, PN-1000493) and suspended in individual droplets. Approximately 10,000 nuclei per sample were sent through the 10X platform for sequencing. This pool was subjected to 2 × 151 bp (paired-end) sequencing according to the manufacturer’s protocol (Illumina NovaSeq). BCL Convert Conversion Software v4.0 (Illumina) was used to generate demultiplexed Fastq files. Reads were mapped to transcriptome mm10 with the pipeline cellranger-7.1.0 (10X Genomics).

Cell Ranger output files were processed using Seurat (v5.2.1) in R (https://cran.r-project.org/web/packages/Seurat/index.html). Raw feature-barcode matrices were imported with Read10X_h5. Seurat objects were constructed with a minimum cell threshold of 2 (min.cells = 2). Quality control was performed for each replicate independently by visualizing the number of detected features per cell (nFeature_RNA) using violin plots and then subsetting cells with low and high feature counts (min/max thresholds ranged from 300–500 and 4000 per sample, respectively). Mitochondrial gene expression was calculated (PercentageFeatureSet, patter*n* = “^mt-”) and cells with >10% mitochondrial transcripts were excluded from downstream analysis.

Each replicate was normalized independently with Seurat’s NormalizeData function. Highly variable features were identified using the FindVariableFeatures method with the “vst” selection approach, retaining the top 2000 features per sample. All genes were scaled with ScaleData, and PCA was performed on variable features for dimensionality reduction. The number of principal components retained was determined based on elbow plots.

For each sample, the FindNeighbors and FindClusters functions were used to construct cell neighbor graphs and identify clusters (resolutio*n* = 0.1), based on the top 15 principal components. Uniform Manifold Approximation and Projection (UMAP) was performed using RunUMAP (dims 1–15) for visualization of cluster structure. Metadata specifying replicate, genotype, and sample identifiers was appended to each Seurat object.

Replicates were then merged and split using Seurat’s SplitObject, followed by normalization, variable feature selection (FindVariableFeatures), scaling (ScaleData), and PCA (RunPCA) analyses. Integration features were identified (SelectIntegrationFeatures), and reciprocal PCA integration was performed using FindIntegrationAnchors (reduction = “rpca”, dims = 1:30). Data across all replicates was integrated with IntegrateData. The resulting integrated object was scaled, and PCA and UMAP were rerun. Clustering was performed with FindNeighbors (dims = 1:20) and FindClusters (resolutio*n* = 0.3).

Cell type scoring was performed by calculating gene signature module scores (AddModuleScore) for relevant cell populations, using marker lists from Petrany et al. (34) The list of cluster defining genes for each population (Supplemental Table 3) was generated by identifying the genes for which log_2_(fold change) of expression in the cluster versus global expression in all nuclei was greater than 0.3.

Proportional representation of clusters by sample and genotype was quantified using the propeller function from the speckle package (v1.4.0) (https://www.bioconductor.org/packages/release/bioc/html/speckle.html). This assessed cluster frequency changes between genotypes. Transformed cluster proportions were calculated with getTransformedProps for further statistical testing. Results were exported for downstream analysis and visualization.

### Bulk RNA-seq

RNA was isolated from flash-frozen TA by use of a phenol/chloroform extraction protocol. Tissue was minced with surgical scissors and homogenized with an OMNI homogenizer in 1 mL TRIzol (Invitrogen, 15596-026) per sample, with total RNA then extracted with chloroform and isopropanol. The RNA pellet was resuspended in 20–35 μL RNase-free water and processed with the RNeasy MinElute Cleanup Kit (Qiagen, 74204). RNA (500 ng total per sample), measured by NanoDrop, was used for sequencing. Library prep and next-generation sequencing were carried out by the Advanced Genomics Core at the University of Michigan. This pool was subjected to 151-bp paired-end sequencing according to the manufacturer’s protocol (Illumina NovaSeqXPlus, System Suite version 1.3.0.39308). BCL Convert Conversion Software v4.0 (Illumina) was used to generate demultiplexed Fastq files. Reads were trimmed using Cutadapt v4.8 (61). FastQC v0.11.8 was used to ensure the quality of data (62). Fastq Screen v0.15.3 was used to screen for various types of contamination (63). Reads were mapped to the reference genome GRCm38 (ENSEMBL), using STAR v2.7.8a (64), and assigned count estimates to genes with RSEM v1.3.3 (65). Alignment options followed ENCODE standards for RNA-seq. QC metrics from several different steps in the pipeline were aggregated by multiQC v1.20 (66). The resulting expected count matrix was then input into DESeq2 for differential expression analysis by R v4.4.3 (67), and thresholds of *P*adj less than 0.05 (as determined by the Wald test with Benjamini-Hochberg correction) and |log_2_(FC)| greater than 0.5849 were used for statistical and biological magnitude significance, respectively. For pathway analysis, iPathwayGuide from Advaita Corporation (68) was used (version v2201, Pathways version release 100.0+/11-12, Nov 21). GSEA was performed with the R library fgsea (1.32.4) (69). Biological pathways for use in GSEA, including PPARGC1A_TARGET_GENES, were obtained from the Gene Transcription Regulation Database (m3.gtrd.v2022.1). GO term analysis was performed using ToppGene (70). The R libraries DESeq2 (1.46.0) (35), ggplot2 (3.5.2) (71), vsn (3.74.0) (72), pheatmap (1.0.13) (73), heatmaply (1.5.0) (74), edgeR (4.4.2) (75), UpSetR (1.4.0) (76, 77), tidyverse (2.0.0) (78), RColorBrewer (1.1.3) (79), dplyr (1.1.4) (80), ggsci (3.2.0) (81), and ggpubr (0.6.0) (82) were used for analysis and data visualization, as well as Prism 10 (GraphPad). GSEA was performed by the Bioinformatics Core of the University of Michigan Medical School’s Biomedical Research Core Facilities (RRID:SCR_019168).

### qPCR

RNA was isolated from frozen tissue as above. cDNA was generated with the High Capacity Reverse Transcription Kit (Applied Biosystems, 4368814) per the manufacturer’s instructions using 2000 ng RNA per sample. qPCR was performed with the use of a 7500 Real-Time PCR SDS System (Applied Biosystems), FastStart TaqMan Probe Master Mix (Roche, 04913957001), and the appropriate primer/probe mix (labeled with FAM) for the gene of interest. Gene expression was compared to *Cpsf2* (labeled with VIC) as a housekeeping gene, multiplexed within the same well. The following TaqMan probes (Thermo Fisher Scientific; product ID in parentheses) were used: *Nmrk2* (Mm01172899_g1), *Clec2d* (Mm00474134_m1), *Slc2a4* (Mm00436615_m1), *Ankrd23* (Mm00463265_m1), *Smyd2* (Mm00660598_m1), *Atp1b1* (Mm00437612_m1), *Ppargc1a* (Mm01208835_m1), *Linc-md1* (Mm03961105_s1), and *Cpsf2* (Mm00489754_m1).

### Western blot

Tissue was minced and homogenized in RIPA Lysis and Extraction Buffer (G-Biosciences, 786-490) with cOmplete Mini protease inhibitor (Roche, 46264500) and centrifuged at 15,000*g* for 15 minutes. Soluble protein in the supernatant was quantified using DC-Assay (Bio-Rad, 5000112). Gels were run with equal amounts of protein in each well in a NuPAGE Bis-Tris Mini Protein Gel, 4%–12% (Invitrogen, NP0335BOX) for 1.5–2 hours at 130 V in 1× NuPAGE MOPS SDS running buffer (Invitrogen, NP0001). Protein was transferred to a PVDF membrane (Merck Millipore, IPVH00010) for 2 hours at 13 V in a semidry transfer apparatus, which was then blocked in 5% milk at room temperature for 30–60 minutes and incubated in primary antibody at 4°C overnight. Membranes were incubated in secondary antibody for 1 hour at room temperature. Protein was visualized using either Pierce ECL Western Blotting Substrate (Thermo Fisher Scientific, 32106) or SuperSignal West Pico PLUS Chemiluminescent Substrate (Thermo Fisher Scientific, 34577). Imaging was performed with an iBright FL1500 system (Invitrogen, A44241). Band intensity was quantified with Fiji (60), with membrane background subtracted, and bands were normalized to the indicated loading control.

### ChIP-seq

TA totaling roughly 200 mg pooled tissue per genotype (7 TA from AR113Q and 4 TA from WT) was collected. Following collection, tissue was shipped to Active Motif Services for chromatin preparation, ChIP reactions, generation of libraries, sequencing of libraries, and ChIP-seq analysis. In brief, tissue was pulverized with mortar and pestle in liquid nitrogen and then fixed in PBS with 1% formaldehyde at room temperature for 15 minutes. Fixation was stopped by the addition of 0.125 M glycine (final). Chromatin was isolated by adding lysis buffer and sonicating using the PIXUL Multi-Sample Sonicator (Active Motif, 53130) to shear DNA to an average fragment size of 200–1000 bp. To determine chromatin yield, an aliquot of the sheared chromatin was reverse crosslinked at 65°C, treated with RNase and proteinase K, and subjected to DNA purification using SPRI beads (Beckman Coulter). DNA concentrations were measured using a Qubit Fluorometer (Thermo Fisher Scientific), and total chromatin yield was extrapolated based on the original chromatin volume.

For ChIP reactions, aliquots of chromatin were precleared with protein G agarose beads (Invitrogen). Immunoprecipitations were performed using an antibody specific for PGC-1α. After washing, immune complexes were eluted from the beads using SDS buffer, treated with RNase and proteinase K, and decrosslinked by overnight incubation at 65°C. ChIP DNA was then purified using phenol/chloroform extraction and ethanol precipitation.

ChIP DNA libraries were prepared using either the PrepX DNA Library Kit (Takara Bio) on the Apollo automation platform or the NEB DNA Library Prep Kit, following the manufacturers’ protocols. Libraries were sequenced on the Illumina platform and the resulting data were analyzed using standard ChIP-seq workflows.

Reads were aligned to the mouse genome (mm10) using the BWA algorithm (v0.7.17, default settings) (https://github.com/lh3/bwa/releases/tag/v0.7.17). Duplicate reads were removed; only uniquely mapped reads were used for further analysis. Alignments were normalized using the counts per million (CPM) method, and the resulting histograms (genomic “signal maps”) were stored as BigWig files. Peaks were identified using the MACS (v2.2.7) (https://pypi.org/project/MACS2/) algorithm at a cutoff of a *q* value of 0.1. Peaks that were on the ENCODE blacklist were removed. Signal maps and peak locations were used as input data to Active Motif’s proprietary analysis program, which creates Microsoft Excel tables containing detailed information on sample comparison, peak metrics, peak locations, and gene annotations.

For downstream analysis, ChIP-seq peaks indicating sites of differential binding were identified using edgeR (thresholds of FDR < 0.05 and |log_2_[FC]| > 1.0) (https://bioconductor.org/packages/release/bioc/html/edgeR.html). Results were filtered to only include differential binding within ±5 kb of a TSS. For genes that had multiple sites of differential binding, the closest peak to the TSS was kept.

### Antibodies

#### Primary antibodies.

The following primary antibodies (antigen, dilution/concentration, vendor, catalog number) were used for these studies: Myh4, 1:100, Developmental Studies Hybridoma Bank at the University of Iowa (DSHB), BF-F3; Myh1, 1:10, DSHB, 6H1; PGC-1α (ChIP-seq), Novus, NBP1-04676; PGC-1α (Western blot), 1:1000, Abcam, ab191838; Vinculin, 1:10,000, Sigma-Aldrich, V9131; MEF2A/C, 1:100, Abcam, ab197070; CBP, 1:200, Invitrogen, PA5-27369; CREB, 1:500, Cell Signaling Technology, D76D11; p62, 1:400, PROGEN, GP62-C; PG-21 (AR), 1:500, Millipore, 06-680.

#### Secondary antibodies.

The following secondary antibodies (antigen, dilution/concentration, vendor, catalog number) were used for these studies: Alexa Fluor 488 goat anti-mouse IgM, 1:300, Invitrogen, A-21042; goat anti-rabbit IgG (H+L)-HRP conjugate, 1:2,000, Bio-Rad, 170-6515; goat anti-mouse IgG (H+L)-HRP conjugate, 1:2,000, Bio-Rad, 170-6516; Alexa Fluor 594 goat anti-rabbit, 1:1000, Invitrogen, A-32740; Alexa Fluor 647 goat anti-guinea pig, 1:1000, Invitrogen, A-21450.

#### Other.

Wheat germ agglutinin–Alexa Fluor Plus 405, 5 μg/mL, Thermo Fisher Scientific, W56132.

### Statistics

Statistical analysis was performed in GraphPad Prism 10 using 2-tailed unpaired Student’s *t* test with Welch’s correction for comparisons of 2 groups and 2-way ANOVA with Tukey’s multiple-comparison test for groups of 3; α less than 0.05 was set as the threshold for significance. Two-sided Fisher’s exact test was used for contingency analysis (Figure 4F). For differential expression analysis by DESeq2, the Wald test was used for statistical significance and the threshold of statistical significance was *P*adj less than 0.05.

### Study approval

All procedures involving AR113Q, AR21Q, and corresponding WT mice were approved by the University of Michigan Committee on Use and Care of Animals (PRO00011667). For R6/2 and corresponding WT mice, animal care and procedures were performed in compliance with United Kingdom Home Office regulations (Animals and Scientific Procedures Act 1986) and were approved by the University College London Ethical Review Process Committee. For SCA1 Q154 and corresponding WT mice, all procedures were approved by the Yale University Institutional Animal Care and Use Committee.

### Data availability

The data discussed in this publication have been deposited in NCBI’s Gene Expression Omnibus under GEO Series accession numbers GSE305394 (ChIP-seq), GSE305395 (AR113Q bulk RNA-seq), GSE305396 (snRNA-seq), and GSE329198 (R6/2 and SCA1 Q154 bulk RNA-seq). Values for all data points associated with figures in the main text and Supplemental figures are available in the accompanying Supporting Data Values file.

## Author contributions

Conceptualization: CJK, LBC, JL, APL. Investigation: CJK, LBC, ZY, LN. Funding acquisition: JL, APL. Supervision: JL, APL. Provision of unique reagent: HTZ. Analyzing data: CJK, LBC. Writing: CJK, APL. Review and editing: CJK, LBC, ZY, LN, HTZ, JL, APL.

## Conflict of interest

HTZ is a full-time employee and shareholder of Ionis Pharmaceuticals.

## Funding support

This work is the result of NIH funding, in whole or in part, and is subject to the NIH Public Access Policy. Through acceptance of this federal funding, the NIH has been given a right to make the work publicly available in PubMed Central.

- NIH grants R01 NS119873 (to APL), R01 AG076154 (to JL), and T32 GM145470 and T32 GM007863 (to CJK).

## Supplementary Material

Supplemental data

Unedited blot and gel images

Supplemental table 1

Supplemental table 2

Supplemental table 3

Supplemental table 4

Supplemental table 5

Supplemental table 6

Supplemental table 7

Supplemental table 8

Supporting data values

## Version 1 05/07/2026

In-Press Preview

## Version 2 07/22/2026

Electronic publication
