# Supplementary material for: PGC-1α pathway dysregulation disrupts myofiber specification in a mouse model of SBMA
Source: JCI Insight. 2026 May 7;11(14):e203215. doi: 10.1172/jci.insight.203215 (PMC13461168; doi:10.1172/jci.insight.203215)
Supplement: Supplementary file 1 [file jciinsight-11-203215-s327.pdf]

Supplemental Material

PGC-1α pathway dysregulation disrupts myofiber specification in a mouse model of SBMA

Supplementary Methods

Intranuclear localization of PGC-1α in PC12 cells

PC12 cells expressing polyQ AR (AR112Q) were generated as described previously (1) and seeded on glass slides pre-coated with poly-D-lysine (MilliporeSigma, A-003-E) diluted to 0.1 mg/mL in PBS. Cells were cultured in high-glucose phenol-red-free DMEM (Gibco, 21063-029) containing 10% charcoal-stripped horse serum (Gibco, 16050-122), 5% charcoal-stripped FBS (Sigma, F0926), 100 units/ml penicillin/streptomycin/glutamine (Invitrogen, 10378016), 200 μg/ml hygromycin B (Invitrogen, 10687010), and 100 μg/ml G418 (Gibco, 10131-035) at 37°C in 10% CO_2. Expression and nuclear translocation of AR were induced by addition of 1 μg/mL doxycycline (Clontech/Takara, 631311) in DMSO and 10 nM R1881 (Sigma, R0908) in ethanol for 3 days, after which cells were fixed upon replacement of media with ice-cold methanol for 4 minutes. Cells were blocked for one hour at room temperature with 10% normal goat serum in PBS with 0.1% Triton X-100. Primary antibodies were added for overnight staining at 4°C, followed by three washes with 0.1% Triton X-100 in PBS and incubation with secondary antibodies for one hour at room temperature in blocking solution. Samples were again washed three times with 0.1% Triton X-100 in PBS and mounted with VECTASHIELD Antifade Mounting Medium with DAPI (Vector Laboratories, H-1200-10). Slides were imaged using a Nikon A1R confocal microscope, and adjustments to brightness and/or contrast were applied equally across entire images using Fiji.

Treatment with 15-prostaglandin dehydrogenase inhibitor

AR113Q mice were treated once daily for 14 days via intraperitoneal injection of either vehicle (10% ethanol, 5% Cremophor EL (Sigma, C5135), and 85% D5W [5% (w/v) dextrose in water]) or 5 mg/kg SW033291 (Selleck, S7900) dissolved in vehicle. Animals were then euthanized for extraction of RNA from tibialis anterior and subsequent measurement of relative gene expression by qPCR, as described in the methods in the main text. The following TaqMan probes (Thermo Fisher Scientific; product ID in parentheses) were used: Nav2 (Mm00614138_m1), Kcnn3 (Mm00446516_m1), Runx1 (Mm01213404_m1), Ncam1 (Mm01149710_m1), Myom3 (Mm01193371_m1), Linc-md1 (Mm03961105_s1), Gadd45a (Mm00432802_m1), Shroom3 (Mm00497207_m1), Hpgd (Mm00515121_m1).

Antibodies

For immunofluorescence, the following primary antibodies (antigen, dilution/concentration, vendor, catalog number) were used: MEF2A/C, 1:100, Abcam, ab197070; CBP, 1:200, Invitrogen, PA5-27369; CREB, 1:500, Cell Signaling Technology, D76D11; p62, 1:400, PROGEN, GP62-C; PGC-1α, 1:200, Millipore, ST1202. The following secondary antibodies were used: AlexaFluor 488 goat anti-mouse, 1:1000, Invitrogen, A-11029; AlexaFluor 647 goat anti-rabbit, 1:1000, Invitrogen, A-21244.

For Western blot of 15-PGDH, the following antibodies (antigen, dilution/concentration, vendor, catalog number) were used: 15-PGDH, 1:1,000, Santa

Cruz Biotechnology, sc-271418; Vinculin, 1:10,000, Sigma Aldrich, V9131; Goat anti-mouse IgG (H+L)-HRP conjugate, 1:2,000, Bio-Rad, 170-6516.

Supplementary References

1. J. L. Walcott, D. E. Merry, Ligand Promotes Intranuclear Inclusions in a Novel Cell Model of Spinal and Bulbar Muscular Atrophy. *J. Biol. Chem.* **277**, 50855–50859 (2002).

Supplementary Tables

Supplementary Table 1. Quality control for snRNA-seq analysis.

This table shows the results of quality control of snRNA-seq analysis in Figure 1.

Supplementary Table 2. Statistically significant cluster population differences by genotype in snRNA-seq.

This table lists statistics for nucleus population difference between genotypes in Figure 1.

Supplementary Table 3. Cluster defining genes for nucleus populations found by snRNA-seq.

This table lists the top 20 cluster defining genes for each nucleus population found by snRNA-seq in Figure 1, as well as all cluster defining genes for each population in separate tabs.

Supplementary Table 4. DESeq2 results and cluster defining gene annotations for AR113Q vs. WT and ASO vs. vehicle bulk RNA-seq at 52 weeks.

This table lists the results from DESeq2 on the comparisons of AR113Q vs. WT and ASO vs. vehicle bulk RNA-sequencing at 52 weeks in separate tabs, along with annotations

indicating presence of each given gene in the cluster defining gene sets for Types IIb, IIb-2, IIx, and 113Q-myo, used for Figure 3.

Supplementary Table 5. GSEA results for AR113Q vs. WT and ASO vs. vehicle bulk RNA-seq at 52 weeks.

This table lists the results of gene set enrichment analysis (GSEA) on the two DESeq2 analyses from the 52-week TA bulk RNA-seq experiment from Figure 3.

Supplementary Table 6. DESeq2 results for AR113Q vs. WT at 5 weeks and 26 weeks.

This table lists the results from DESeq2 on the comparison of AR113Q vs. WT at 5 weeks and 26 weeks in separate tabs, used for Supplementary Figure 3.

Supplementary Table 7. DESeq2 results for R6/2 transgenic and SCA1 Q154 bulk RNA-seq.

This table lists the results from DESeq2 on the comparison of disease vs. age-, sex- and tissue-matched controls for R6/2 transgenic (12 weeks, male, quadriceps) and SCA1 Q154 (24 weeks, male, TA) in separate tabs, used for Figure 5 and Supplementary Figure 8.

Supplementary Table 8. GO term analysis of genes with decreased chromatin binding and decreased expression.

This table lists the results of gene ontology (GO) term analysis from the genes in the decreased chromatin binding-decreased gene expression quadrant in Figure 6E.

Supplementary Figures

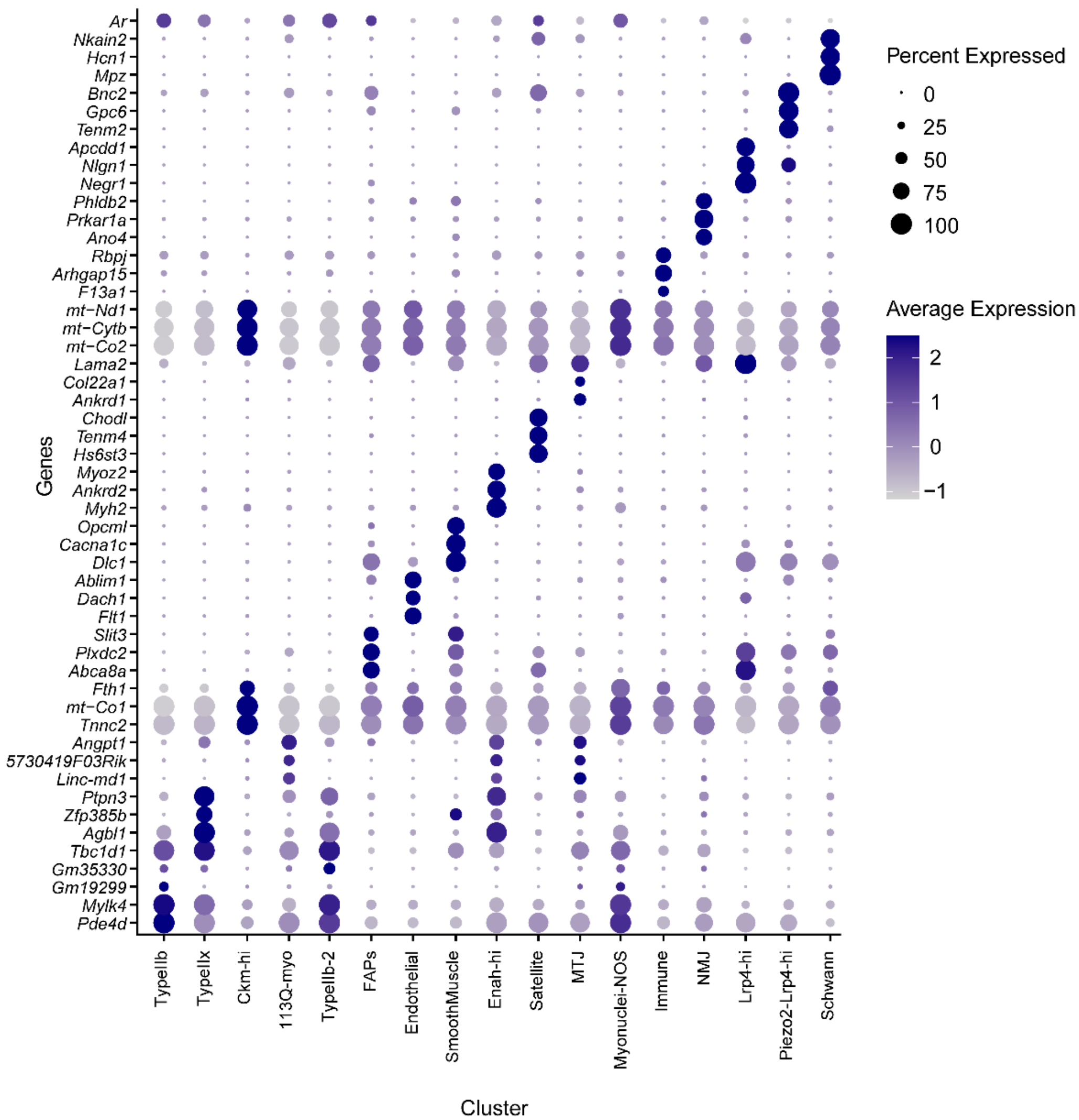

Supplementary Figure 1. Bubble matrix of cluster defining genes from snRNA-seq.

Bubble matrix showing top three cluster defining genes for each nucleus cluster in Fig. 1B, with *Ar* at the top. The size of each bubble represents the percentage of nuclei in

each cluster expressing the given gene, while the color of each bubble represents the average expression across the whole cluster.

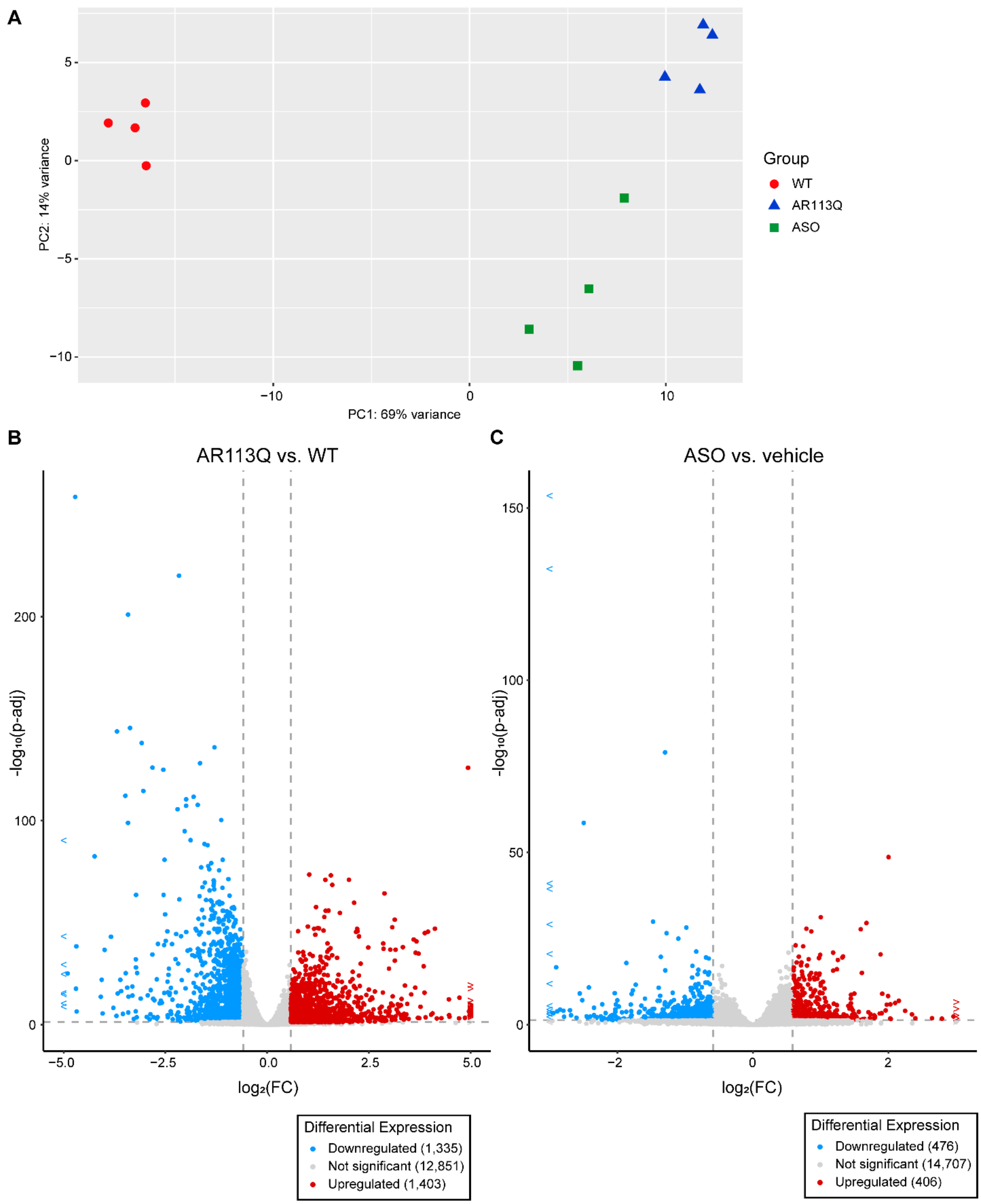

Supplementary Figure 2. Bulk RNA-seq of WT, AR113Q, and AR113Q + ASO mice at 52 weeks.

AR113Q or WT males were administered *AR*-targeted ASO (25 mg/kg body weight) or vehicle subcutaneously, once per week, from 26 to 52 weeks. RNA was extracted from tibialis anterior (TA) at 52 weeks for bulk RNA-sequencing.

A. PCA plot showing samples from bulk RNA-sequencing of vehicle-treated WT mice, vehicle-treated AR113Q mice, and ASO-treated AR113Q mice (n = 4 male mice/group).

B., C. Volcano plots showing results of differential expression analysis by DESeq2 in the comparisons of vehicle-treated AR113Q vs. vehicle-treated WT (B) and ASO-treated AR113Q vs. vehicle-treated AR113Q (C). Dotted lines indicate thresholds of p-adj < 0.05, $|\log_2(FC)| > 0.5849$. Genes meeting both thresholds for statistical and biological significance are indicated in blue (downregulated) and red (upregulated), with all other genes in light gray (not significant). For ease of visualization, < and > indicate out-of-bounds values for $\log_2(FC)$ of a small number of genes (cutoffs of $|\log_2(FC)| > 5$ for (B) and $|\log_2(FC)| > 3$ for (C)); original values available in Supplementary Table 4.

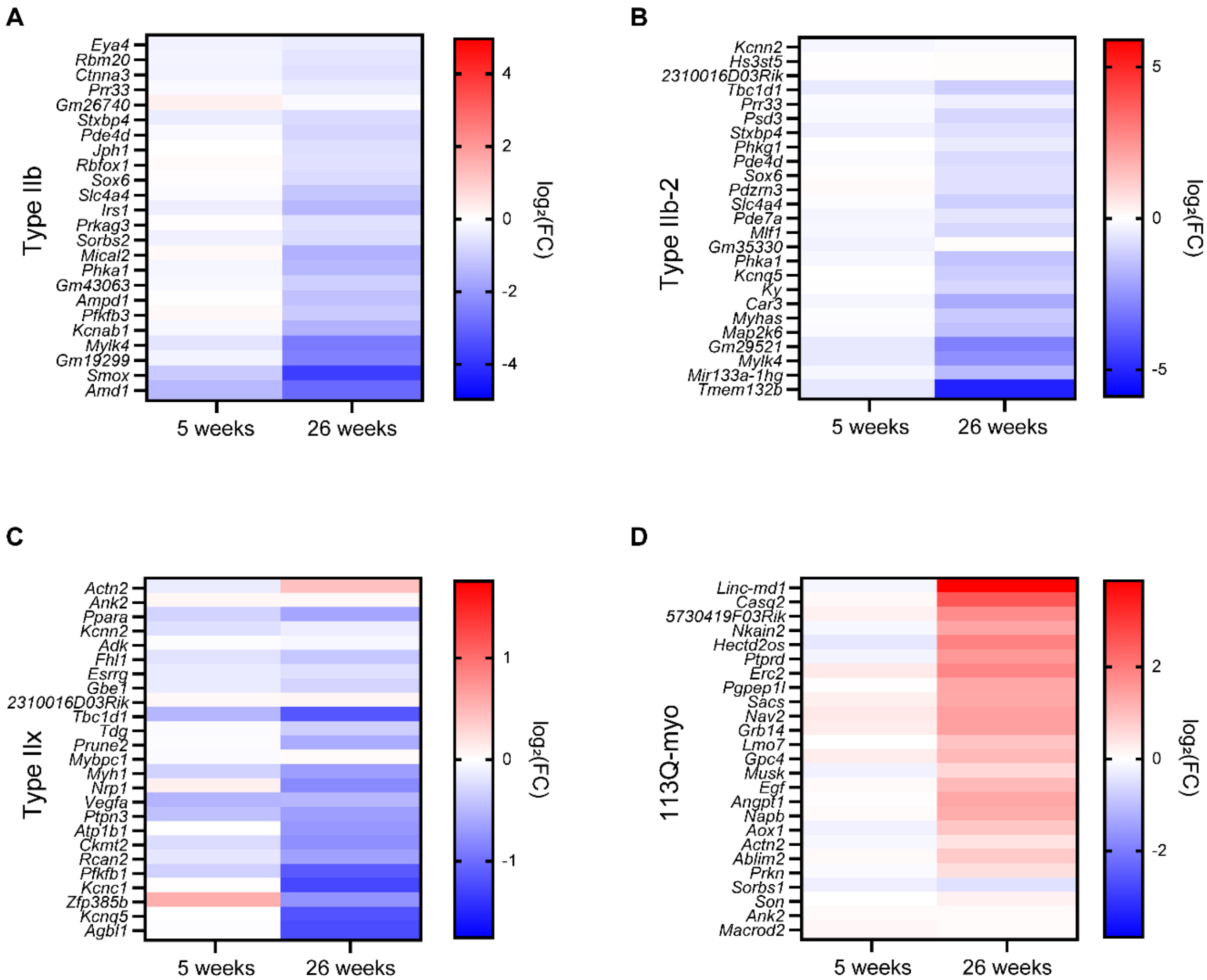

Supplementary Figure 3. Bulk RNA-seq expression of cluster defining genes at 5 and 26 weeks.

A.-D. Heatmaps showing $\log_2$(fold-change), between AR113Q vs. WT, of the top 25 cluster defining genes for each of the indicated myonuclei groups, as assessed by bulk RNA-seq of TA from cohorts of 5-week male mice (n = 3 WT, 4 AR113Q) and 26-week male mice (n = 4/group). Color scales match those of Fig. 3A-D. In (A), the cluster defining gene *Raet1e* was not detected by RNA-sequencing at 5 or 26 weeks, so it was not visualized here.

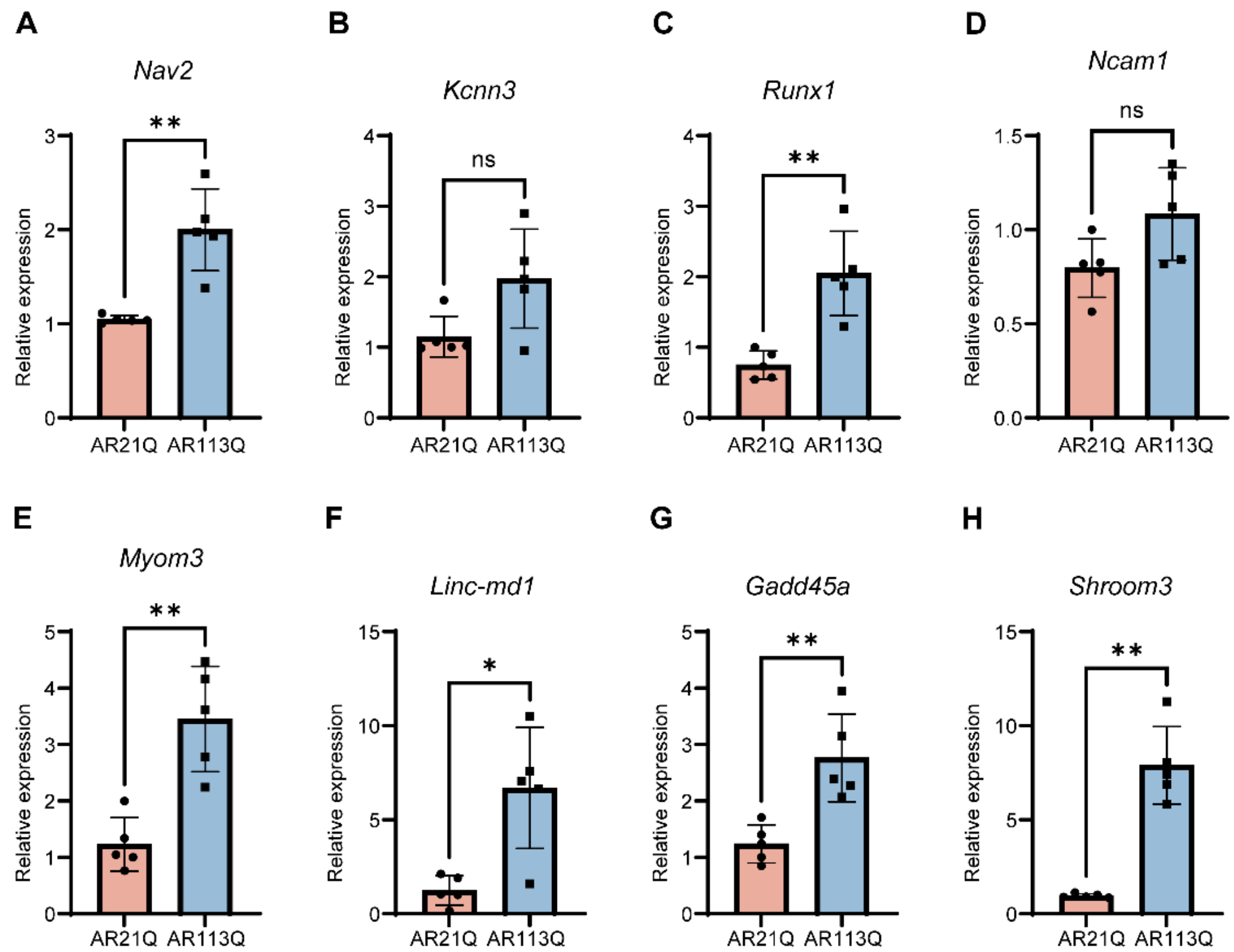

Supplementary Figure 4. Expression of genes of interest from acutely denervated myonuclei in AR113Q muscle.

Relative mRNA expression in the TA from AR21Q or AR113Q male mice at 26 weeks (n = 5/group) was determined by qPCR: *Nav2* (A), *Kcnn3* (B), *Runx1* (C), *Ncam1* (D), *Myom3* (E), *Linc-md1* (F), *Gadd45a* (G), and *Shroom3* (H).

Data are mean ± SD. ns = not significant, $*p < 0.05$, $**p < 0.01$ by two-tailed unpaired *t* test with Welch's correction. (A) t = 4.875; df = 4.071; p = 0.0078. (B) t = 2.424; df = 5.315; p = 0.0569. (C) t = 4.596; df = 4.881; p = 0.0062. (D) t = 2.207; df = 6.768; p = 0.0644. (E) t = 4.746; df = 5.952; p = 0.0032. (F) t = 3.669; df = 4.475; p = 0.0176. (G) t = 4.025; df = 5.456; p = 0.0084. (H) t = 7.497; df = 4.024; p = 0.0017.

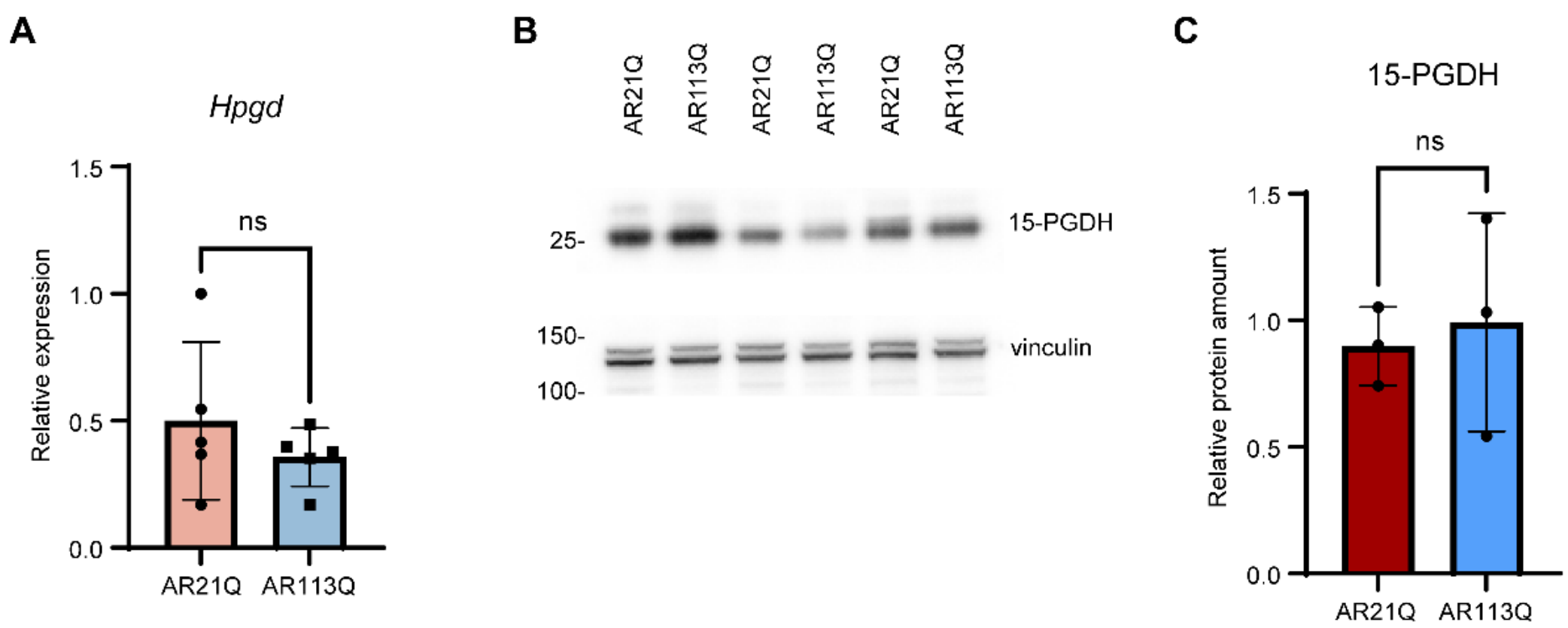

Supplementary Figure 5. 15-prostaglandin dehydrogenase expression is unchanged in AR113Q skeletal muscle.

A. Relative expression levels of *Hpgd*, the gene encoding for 15-PGDH, in TA at 26 weeks from AR21Q and AR113Q male mice (n = 5/group) by qPCR.

B. Western blot of 15-PGDH and vinculin in TA at 26 weeks from AR21Q and AR113Q male mice (n = 3/group).

C. Quantification of 15-PGDH levels in (B), normalized to vinculin.

Data are mean ± SD. ns = not significant by two-tailed unpaired *t* test with Welch's correction. (A) t = 0.9663; df = 5.081; p = 0.3776. (C) t = 0.3566; df = 2.511; p = 0.7492.

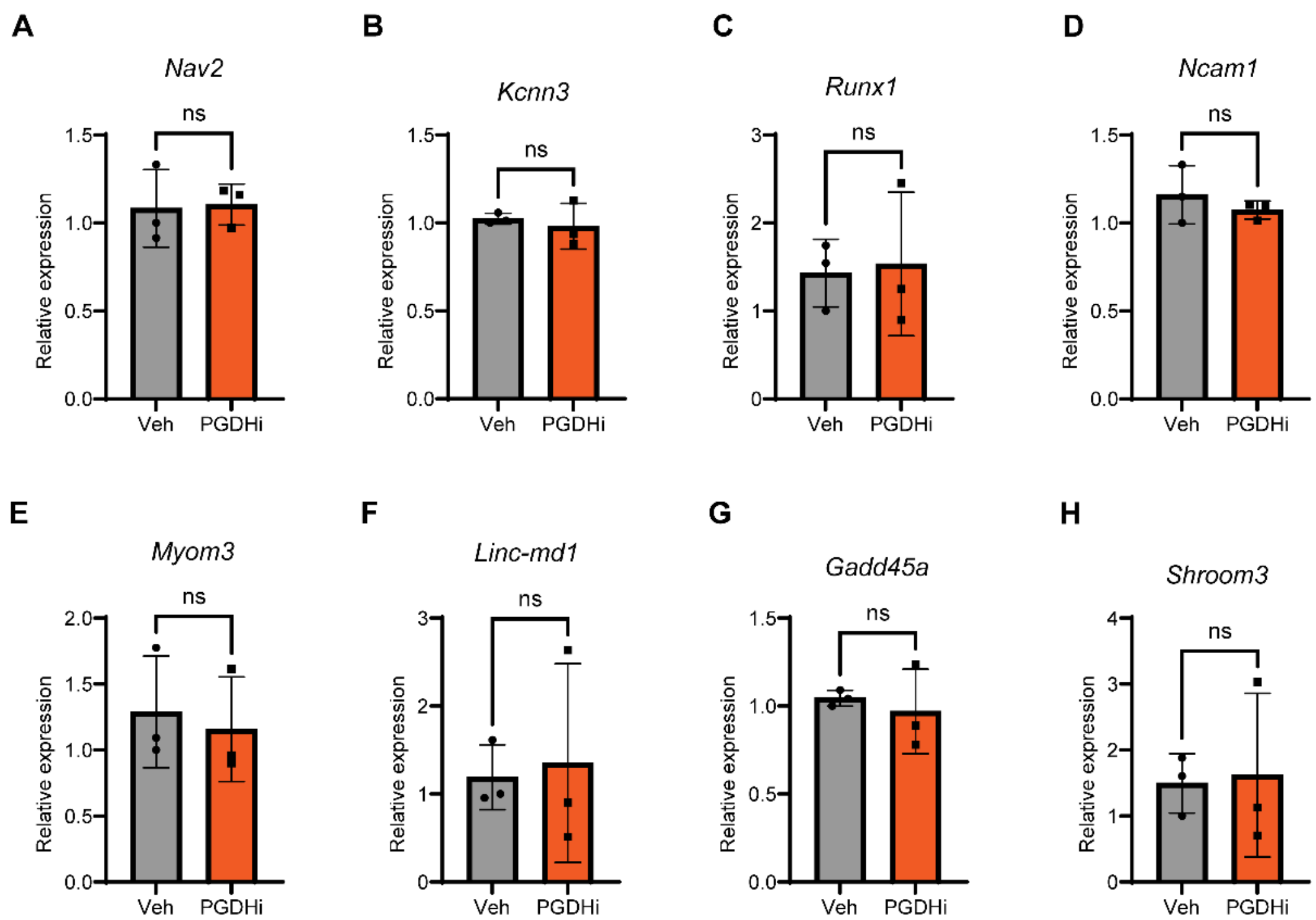

Supplementary Figure 6. Effect of SW033291, an inhibitor of 15-PGDH, on gene expression in AR113Q muscle.

A.-H. AR113Q males were treated by intraperitoneal administration of SW033291 (PGDHi) or vehicle (Veh) daily from 24-26 weeks of age at 5 mg/kg (n = 3/group). Expression levels in TA of the eight target genes from Supplementary Figure 5 were measured by qPCR: *Nav2* (A), *Kcnn3* (B), *Runx1* (C), *Ncam1* (D), *Myom3* (E), *Linc-md1* (F), *Gadd45a* (G), *Shroom3* (H).

Data are mean ± SD. ns = not significant by two-tailed unpaired *t* test with Welch's correction. (A) t = 0.1557; df = 3.032; p = 0.8861. (B) t = 0.5561; df = 2.220; p = 0.6291. (C) t = 0.1988; df = 2.847; p = 0.8558. (D) t = 0.8711; df = 2.382; p = 0.4625. (E) t =

0.3941; df = 3.982; p = 0.7137. (F) t = 0.2355; df = 2.419; p = 0.8323. (G) t = 0.5324; df = 2.144; p = 0.6445. (H) t = 0.1623; df = 2.519; p = 0.8832.

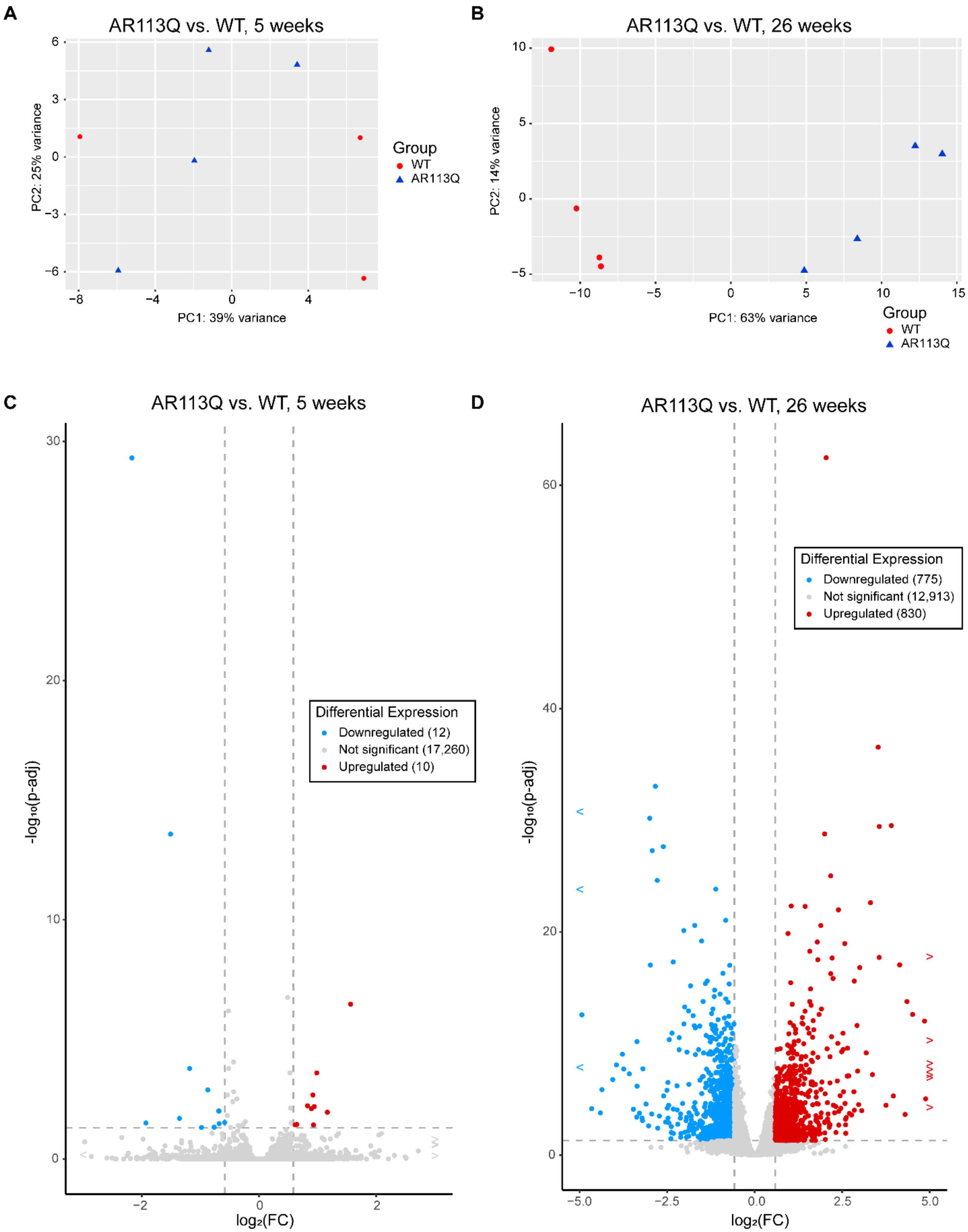

Supplementary Figure 7. Bulk RNA-seq of AR113Q vs. WT at 5 and 26 weeks.

RNA was extracted from TA from male mice at 5 weeks or 26 weeks and analyzed by bulk RNA-sequencing.

A., B. PCA plots showing samples from AR113Q and WT male mice at 5 weeks (A) or 26 weeks (B). n = 3 mice for 5-week WT and 4 mice for all other groups.

C., D. Volcano plots showing results of differential expression analysis by DESeq2 in the comparisons of AR113Q vs. WT at 5 weeks (C) or 26 weeks (D). Dotted lines indicate thresholds of $p\text{-adj} < 0.05$, $|\log_2(FC)| > 0.5849$. Genes meeting both thresholds for statistical and biological significance are indicated in blue (downregulated) and red (upregulated), with all other genes in light gray (not significant). For ease of visualization, < and > indicate out-of-bounds values for $\log_2(FC)$ of a small number of genes (cutoffs of $|\log_2(FC)| > 3$ for (C) and $|\log_2(FC)| > 5$ for (D)); original values available in Supplementary Table 6.

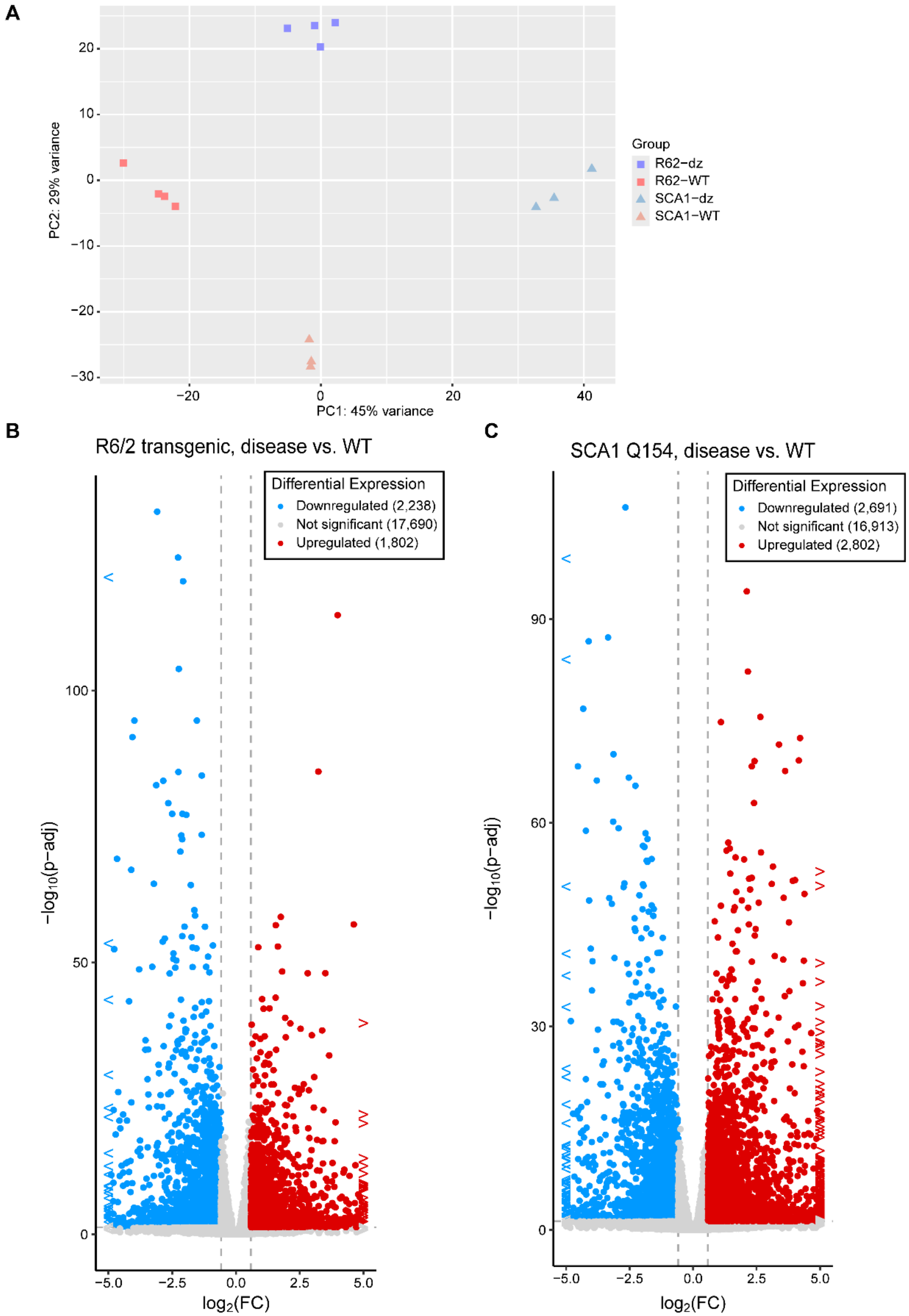
A
PC2: 29% variance
PC1: 45% variance
Group
R62−dz
R62−WT
SCA1−dz
SCA1−WT
B
R6/2 transgenic, disease vs. WT
Differential Expression
Downregulated (2,238)
Not significant (17,690)
Upregulated (1,802)
−log10(p−adj)
log2(FC)
C
SCA1 Q154, disease vs. WT
Differential Expression
Downregulated (2,691)
Not significant (16,913)
Upregulated (2,802)
−log10(p−adj)
log2(FC)

Supplementary Figure 8. Bulk RNA-seq of SCA1 Q154 and R6/2 transgenic skeletal muscle at symptomatic ages.

RNA was extracted from the TA of SCA1 Q154 male mice at 24 weeks and the quadriceps of R6/2 transgenic male mice at 12 weeks and analyzed by bulk RNA-sequencing.

A. PCA plot showing samples from SCA1 Q154 and R6/2 transgenic mice, along with their age-matched WT controls. n = 4 mice for the R6/2 transgenic mice and controls, n = 3 mice for the SCA1 Q154 mice and controls.

B., C. Volcano plots showing results of differential expression analysis by DESeq2 in the comparisons of R6/2 disease vs. WT (B) or SCA1 disease vs. WT (C). Dotted lines indicate thresholds of p-adj < 0.05, $|\log_2(FC)| > 0.5849$. Genes meeting both thresholds for statistical and biological significance are indicated in blue (downregulated) and red (upregulated), with all other genes in light gray (not significant). For ease of visualization, $<$ and $>$ indicate out-of-bounds values for $\log_2(FC)$ of a small number of genes (cutoffs of $|\log_2(FC)| > 3$ for (C) and $|\log_2(FC)| > 5$ for (D)); original values available in Supplementary Table 7.

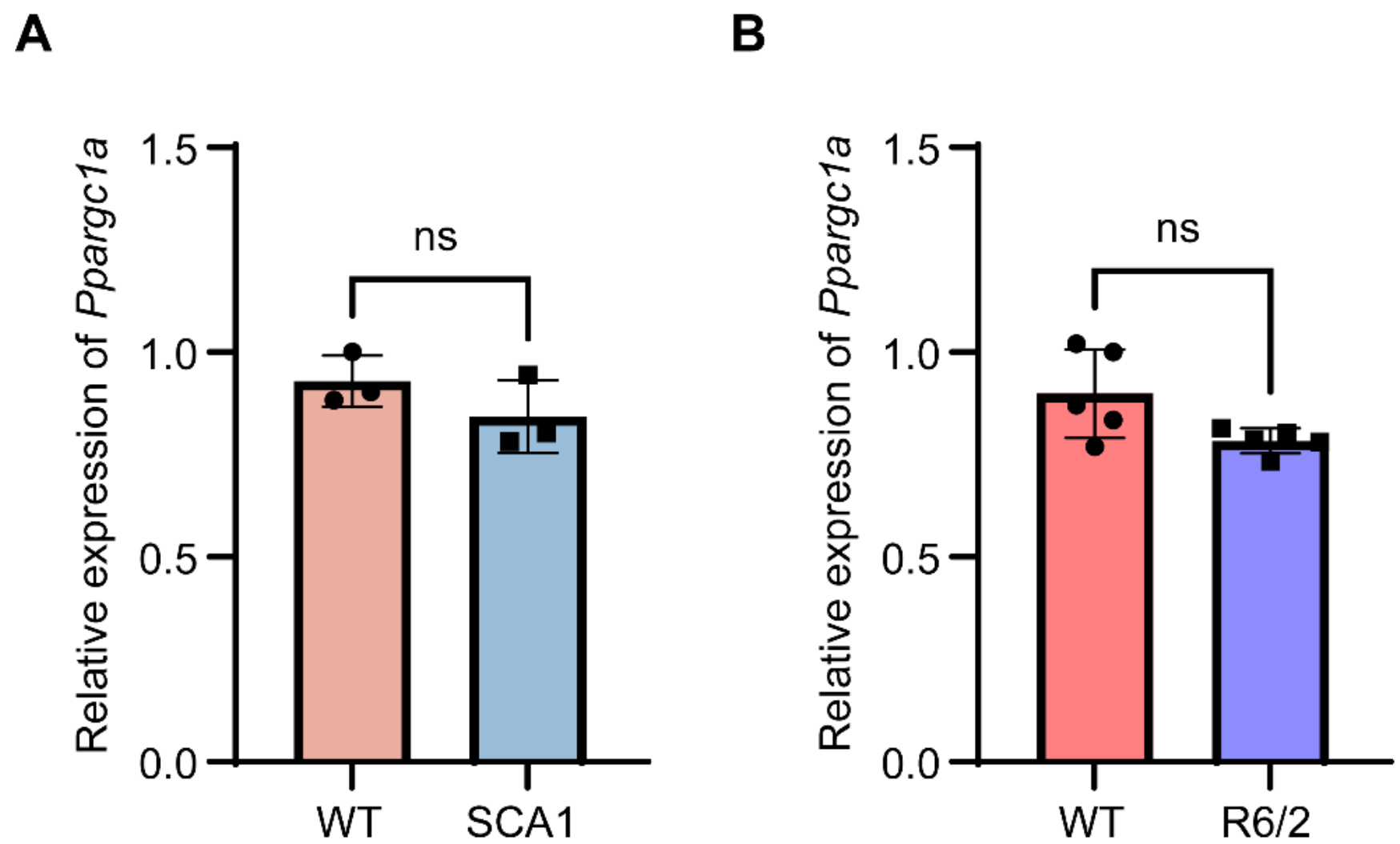

Supplementary Figure 9. *Ppargc1a* expression in SCA1 Q154 and R6/2 muscle.

A., B. Relative gene expression by qPCR of *Ppargc1a* mRNA in TA from SCA1 Q154 knock-in (A, 24 weeks, n = 3 males/group) and quadriceps from R6/2 transgenic (B, 12 weeks, n = 5 males/group) models compared to age- and tissue-matched WT male controls.

Data are mean ± SD. ns, not significant by two-tailed unpaired *t* test with Welch's correction. (A) t = 1.369; df = 3.589; p = 0.2502. (B) t = 2.297; df = 4.642; p = 0.0741.

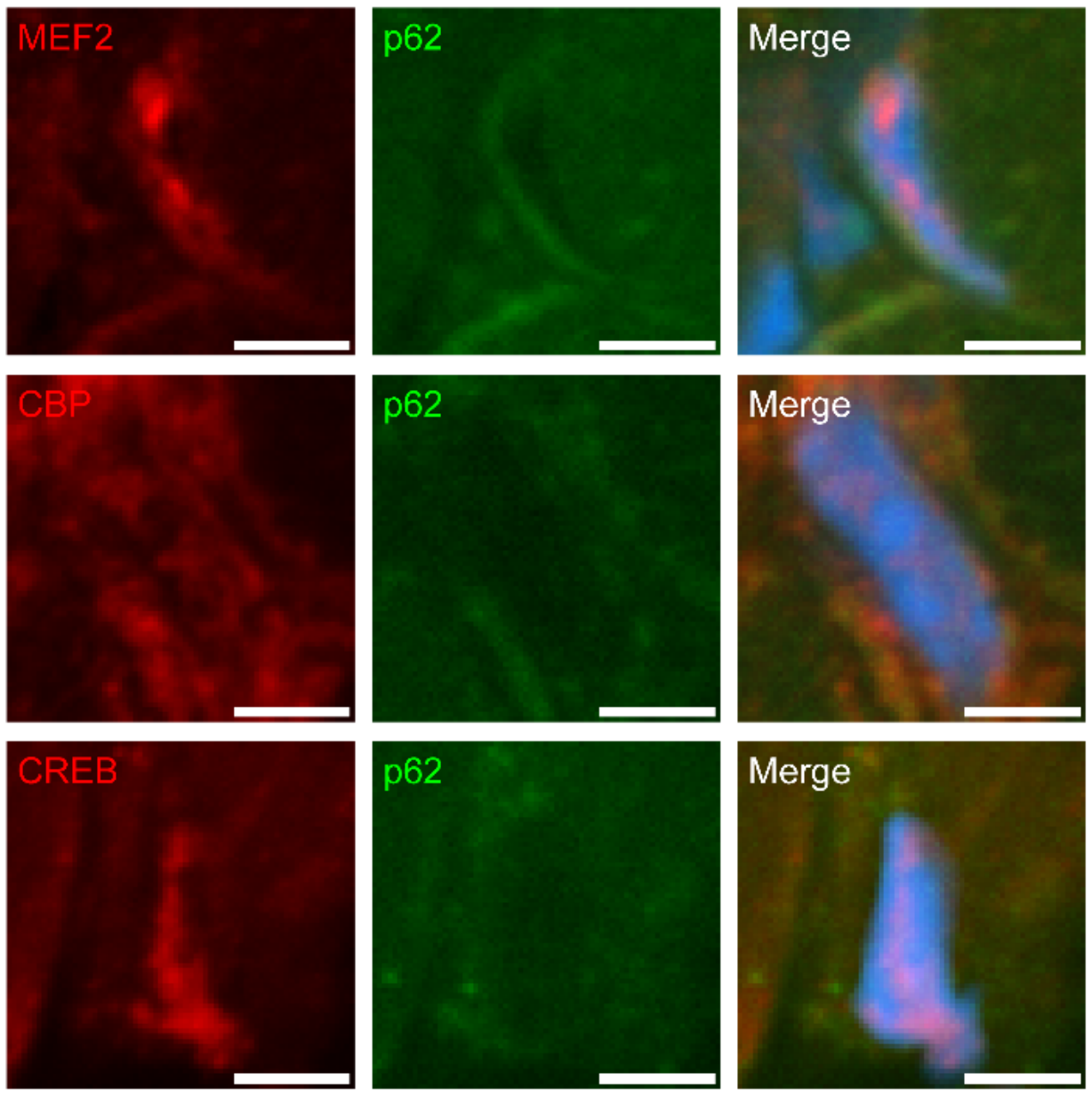

Supplementary Figure 10. MEF2, CBP, and CREB expression in WT muscle.

Representative immunofluorescence images of intranuclear MEF2 (top), CBP (middle), and CREB (bottom) staining in levator ani/bulbocavernosus samples from WT male mice at 26 weeks. Co-stained with p62 (green) and DAPI (blue). Scale bar = 5 μm.

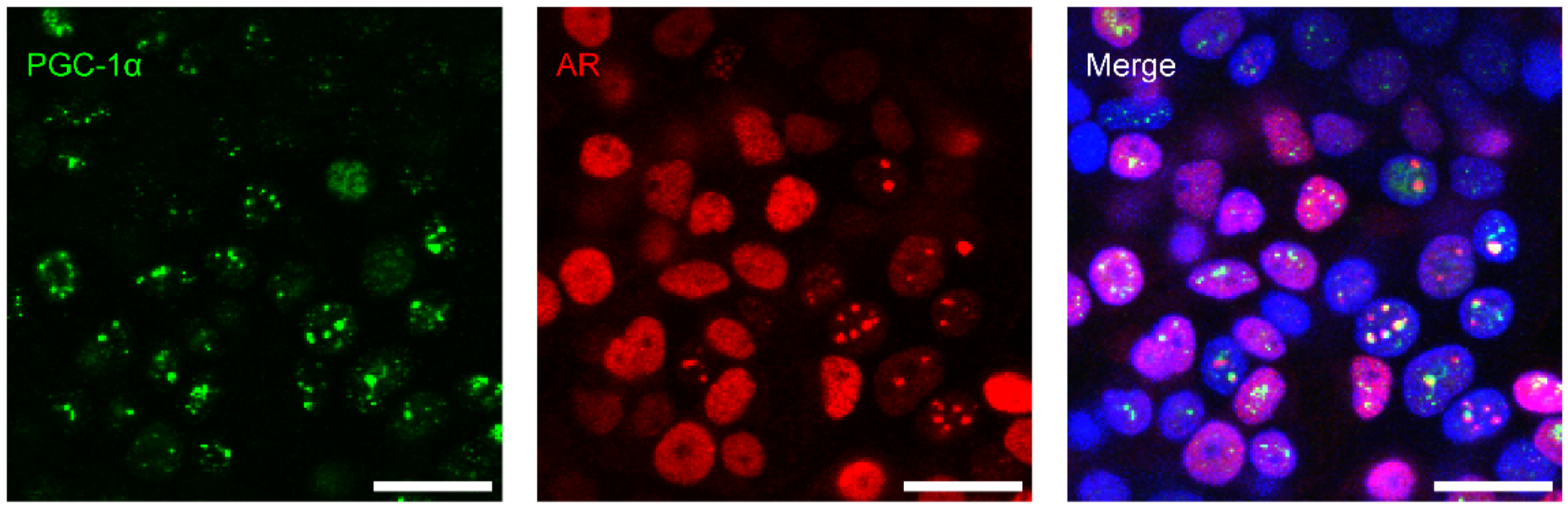

Supplementary Figure 11. Intranuclear distribution of PGC-1α does not differ by polyQ AR aggregation state.

Representative immunofluorescence images of intranuclear PGC-1α (green), AR (red), and DAPI (blue) in PC12 cells expressing tetracycline-regulated polyQ AR treated for three days with doxycycline and 10 nM R1881. Scale bar = 20 µm.

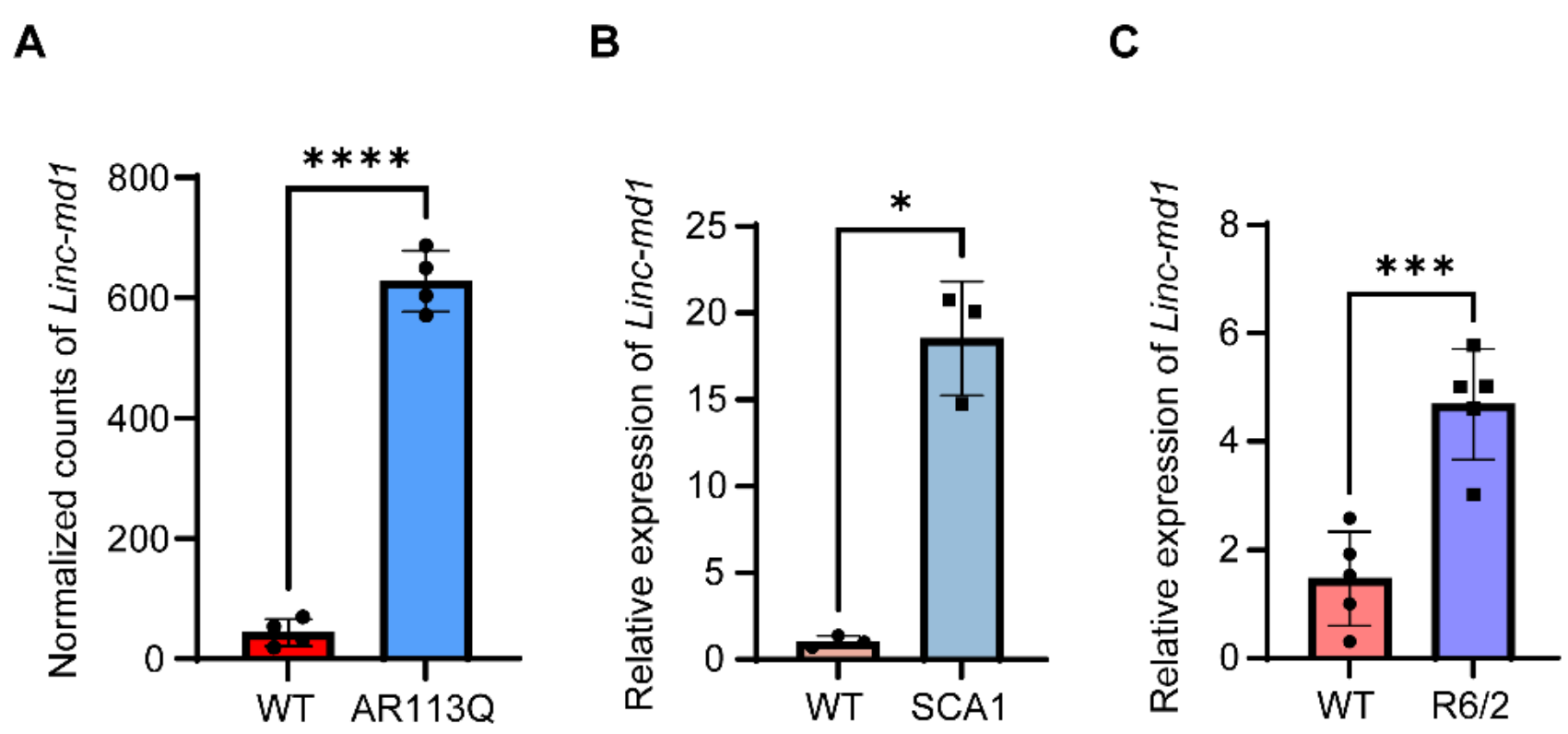

Supplementary Figure 12. ***Linc-md1*** **expression in polyQ muscle.**

A. Normalized counts by DESeq2 of *Linc-md1* in bulk RNA-seq results of TA from WT, AR113Q males at 52 weeks (n = 4/group).

B., C. Relative gene expression by qPCR of *Linc-md1* mRNA in TA from SCA1 Q154 knock-in (B, 24 weeks, n = 3 males/group) and quadriceps from R6/2 transgenic (C, 12 weeks, n = 5 males/group) models compared to age- and tissue-matched WT male controls.

Data are mean ± SD. *$p < 0.05$, ***$p < 0.001$, ****$p < 0.0001$ by two-tailed unpaired *t* test with Welch's correction. (A) $t = 21.01$; $df = 4.117$; $p < 0.0001$. (B) $t = 9.185$; $df = 2.044$; $p = 0.0109$. (C) $t = 5.359$; $df = 7.796$; $p = 0.0007$.
