# Supplementary material for: PGC-1α pathway dysregulation disrupts myofiber specification in a mouse model of SBMA
Source: JCI Insight. 2026 May 7;11(14):e203215. doi: 10.1172/jci.insight.203215 (PMC13461168; doi:10.1172/jci.insight.203215)

Fig. 6b: PGC-1α in TA

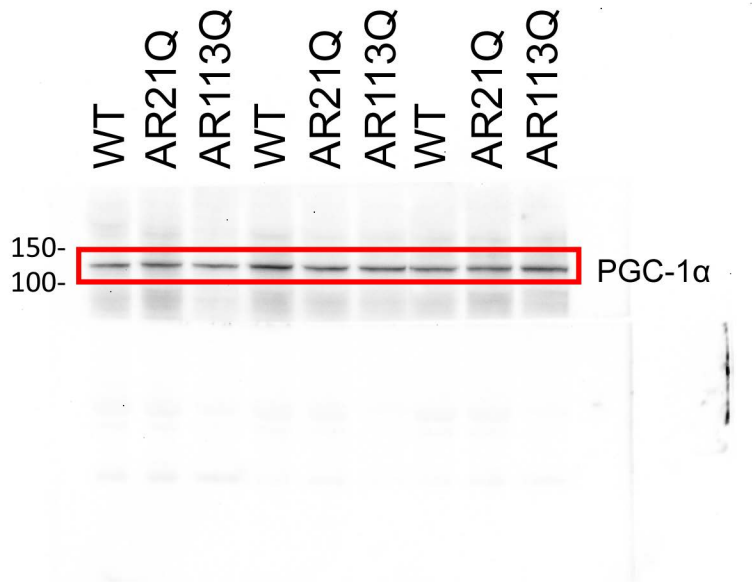

Fig. 6b:
Vinculin in TA

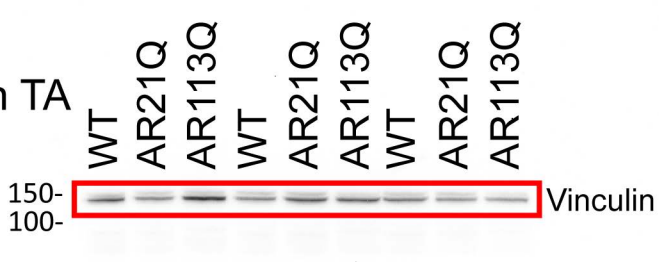

Sup. Fig. 5b: 15-PGDH in TA

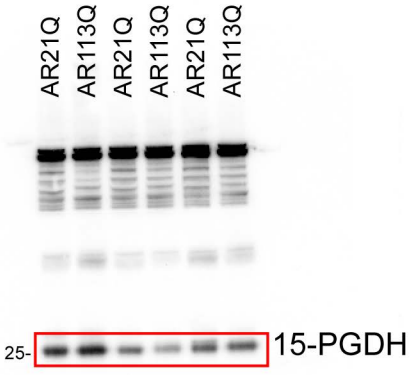

Sup. Fig. 5b: Vinculin in TA

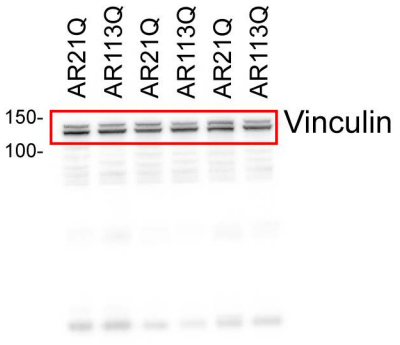

Supplement: Supplementary file 2 [file jciinsight-11-203215-s328.pdf]
